# TGF-β2 Regulates Transcription of the K^+^/Cl^−^ Cotransporter 2 (KCC2) in Immature Neurons and Its Phosphorylation at T1007 in Differentiated Neurons

**DOI:** 10.3390/cells11233861

**Published:** 2022-11-30

**Authors:** Anastasia Rigkou, Attila Magyar, Jan Manuel Speer, Eleni Roussa

**Affiliations:** Institute for Anatomy and Cell Biology, Department of Molecular Embryology, Faculty of Medicine, Albert-Ludwigs-Universität Freiburg, Albertstr. 17, D-79104 Freiburg, Germany

**Keywords:** neurotrophins, GABA, growth factors, chloride homeostasis, pre-Bötzinger complex

## Abstract

KCC2 mediates extrusion of K^+^ and Cl^−^ and assuresthe developmental “switch” in GABA function during neuronal maturation. However, the molecular mechanisms underlying KCC2 regulation are not fully elucidated. We investigated the impact of transforming growth factor beta 2 (TGF-β2) on KCC2 during neuronal maturation using quantitative RT-PCR, immunoblotting, immunofluorescence and chromatin immunoprecipitation in primary mouse hippocampal neurons and brain tissue from *Tgf-β2*-deficient mice. Inhibition of TGF-β/activin signaling downregulates *Kcc2* transcript in immature neurons. In the forebrain of *Tgf-β2*^−/−^ mice, expression of *Kcc2,* transcription factor *Ap2β* and KCC2 protein is downregulated. AP2β binds to *Kcc2* promoter, a binding absent in *Tgf-β2*^−/−^. In hindbrain/brainstem tissue of *Tgf-β2*^−/−^ mice, KCC2 phosphorylation at T1007 is increased and approximately half of pre-Bötzinger-complex neurons lack membrane KCC2 phenotypes rescued through exogenous TGF-β2. These results demonstrate that TGF-β2 regulates KCC2 transcription in immature neurons, possibly acting upstream of AP2β, and contributes to the developmental dephosphorylation of KCC2 at T1007. The present work suggests multiple and divergent roles for TGF-β2 on KCC2 during neuronal maturation and provides novel mechanistic insights for TGF-β2-mediated regulation of KCC2 gene expression, posttranslational modification and surface expression. We propose TGF-β2 as a major regulator of KCC2 with putative implications for pathophysiological conditions.

## 1. Introduction

K^+^/Cl^−^ cotransporter 2 (KCC2; SLC12A5) mediates the electroneutral extrusion of K^+^ and Cl^−^ across neuronal membranes and its developmental upregulation controls the developmental “switch” in GABA function from depolarizing and excitatory in immature neurons to hyperpolarizing and inhibitory in mature neurons [1]. Independent of its transport function, KCC2 regulates the development and morphology of dendritic spines [2,3,4] and is also involved in ontogenetic cell death of cortical neuronal subpopulations [5]. Deficits in KCC2 function significantly contribute to the pathogenesis of several neurological disorders such as epilepsy and neuropathic pain, and can occur following traumatic brain injury [6,7]. Consequently, KCC2 is an attractive target for therapeutic treatment.

Phosphorylation is a potent regulatory mechanism of KCC2 activity with broad pathophysiological implications [7,8,9]. PKC-mediated phosphorylation at S940 promotes KCC2 cell surface stability in vitro, thereby enhancing KCC2 function [10], whereas WNK-regulated phosphorylation at T906/T1007 inhibits KCC2 [11,12,13]. In vivo, knock-in mice expressing homozygous phosphomimetic KCC2 mutations at T906/T1007 die early postnatally [14,15]; in mice with constitutive dephosphorylation of T906/T1007, the onset of seizures is delayed and the severity of seizures attenuated [16], whereas in mice harboring the S940 mutation, both the development and lethality of status epilepticus is accelerated [17].

The two KCC2 isoforms, KCC2a and KCC2b, are generated by alternative splicing of the *Kcc2* gene [18]. *Kcc2* transcription is positively regulated by the transcription factors early growth response 4 (Egr4) and upstream stimulating factors 1 and 2 (USF1, USF2) [19,20] and repressed by neuron-restrictive silencing elements (NRSE) through binding to a NRSF/repressor-element transcription factor (REST) [21,22]. In immature neurons, brain-derived neurotrophic factor (BDNF) and Neurturin have potential to induce *Kcc2* transcription through activation of the signaling cascade ERK1/2/Egr4 [23,24], whereas in mature neurons, BDNF downregulates KCC2 expression [25,26]. Taken together, despite the biological significance of KCC2, data on molecular mechanisms underlying its transcriptional regulation are limited.

The TGF-β family members are signaling molecules that may exert individual, cooperative and synergistic effects in the CNS, thereby regulating several developmental and homeostatic functions including neuronal development, synaptic connectivity and adult neurogenesis (for review, see [27]). TGF-β ligands signal via a heterotetrameric receptor complex composed of TGF-β type II and type I (ALK5) receptors, and may activate the Smad-dependent, canonical and/or non-canonical signaling pathways. During CNS development—although both TGF-β2 and TGF-β3 isoforms are expressed in neural progenitor cells, in radial glia cells and in differentiating neurons—TGF-β2 has been considered relatively more relevant compared to TGF-β3 [28]. Interestingly, several transport proteins have been identified as targets of TGF-β signaling within and outside the CNS, including CFTR, ENaC, distinct V-ATPase subunits in epithelia and NBCe1 in astrocytes [29,30,31,32]. We have previously shown that TGF-β signaling regulates trafficking of KCC2 to the plasma membrane in differentiating and mature mouse hippocampal neurons via TGF-β/CREB/Rab11b pathway [33]. Given the cell-type- and context-dependent actions of TGF-β isoforms and the role of TGF-β2 in regulating neuronal development and synaptic transmission, in the present study, we sought to investigate the effects of TGF-β2 on KCC2 during neuronal maturation. The results show multiple and divergent modes of action for TGF-β2 on KCC2 during neuronal maturation. TGF-β2 positively regulates *Kcc2* expression in immature neurons, possibly acting upstream of the transcription factor AP2β and its binding to the *Kcc2* promoter. Moreover, TGF-β2 contributes to the developmental regulation of the inhibitory phosphorylation of KCC2 at T1007 and, additionally, to the surface expression of KCC2 in neuronal populations of the pre-Bötzinger complex.

## 2. Materials and Methods

### 2.1. Antibodies and Reagents/Chemicals

The following antibodies were used as primary antibodies: anti-KCC2 rabbit polyclonal from Millipore (Cat# 07-432, RRID: AB_310611), anti-phosphorylated KCC2 (S940) rabbit polyclonal from Origene (Cat# TA309219), anti-phosphorylated KCC2 (T1007) rabbit polyclonal from Biomol (Cat# Cay29292) for Western blots, anti-GAPDH mouse monoclonal from Proteintech (Cat# 60004-1-Ig, RRID: AB_2107436), anti-Na^+^/K^+^-ATPase alpha-1 subunit, mouse monoclonal from Developmental Studies Hybridoma bank (Cat# a6F, RRID: AB_528092), anti-Somatostatin (SST) goat polyclonal from Santa Cruz Biotechnology (Cat# sc-7819, RRID: AB_2302603), anti-substance P receptor/neurokinin 1 receptor (NK1R) rabbit polyclonal from Sigma Aldrich (Cat# S8305, RRID: AB_261562) and anti-phox2b rabbit polyclonal from Sigma Aldrich (Cat# P0371, RRID: AB_477302). Phosphorylated KCC2 T906 and T1007 sheep polyclonal antibodies were from the MRC Protein Phosphorylation and Ubiquitination Unit of the University of Dundee: KCC3 phospho T982 (corresponding to KCC2A phospho T906, Sheep# S959C) and KCC3A phospho T1048 (corresponding to KCC2A phospho T1007, Sheep# S961C) for immunocytochemistry. For chromatin immunoprecipitation assay, anti-AP2β rabbit polyclonal was obtained from Cell Signaling Technology (Cat# 2509, RRID: AB_2058198).

For immunofluorescence, donkey anti-rabbit AlexaFluor594 (Cat# 711-585-152, RRID: AB_2340621) and donkey anti-sheep AlexaFluor594 (Cat# 713-585-147, RRID: AB_2340748) were obtained from Jackson ImmunoResearch Labs and donkey anti-goat AlexaFluor488 from Thermo Fisher Scientific (Cat# A-11055, RRID: AB_2534102) were used as secondary antibodies. For Western blots, goat anti-mouse (Cat# 7076, RRID: AB_330924) or anti-rabbit (Cat# 7074, RRID: AB_2099233) IgG coupled to horseradish peroxidase from Cell Signaling Technology were used as secondary antibodies. Human recombinant TGF-β2 (Cat# 302-B2-002/CF) was purchased from R&D Systems and SB431542 (Cat# 1614) was purchased from Tocris.

### 2.2. Animals

All protocols were carried out in accordance with German ethical guidelines for laboratory animals and approved by the Institutional Animal Care and Use Committee of the University of Freiburg and the ethics committee of the City of Freiburg (authorizations: X14/16H, X19/09C and G-21/140). Adult C57BL/6N mice (strain code 027) of either sex were maintained on a 12 h dark/light cycle with food and water ad libitum. Mice were sacrificed by cervical dislocation, and all efforts were made to minimize suffering. Embryos at embryonic day 17.5–18.5 were sacrificed by decapitation and brains were isolated. Subsequently, either hippocampus was excised for generation of primary neuronal cultures, or forebrain and hindbrain/brainstem tissue were separated or acute brainstem slices were cut and processed as described below. *Tgf-β2*-deficient mice have been described earlier [34].

### 2.3. Primary Cultures of Mouse E17.5-18.5 Hippocampal Neurons

Hippocampal neurons were isolated from C57BL/6, *Tgf-β2^+/+^* and *Tgf-β2^−/−^* mice at embryonic (E) day 17.5 or 18.5 of gestation, as described earlier [33]. At day in vitro (DIV) 4, cultures were treated with either recombinant TGF-β2 (2 ng/mL) or 10 µM SB431542, an ALK4/5/7 inhibitor [35], for 60 min, and cells were subsequently processed for either quantitative RT-PCR, immunoblotting or immunocytochemistry.

### 2.4. Quantitative RT-PCR

Total RNA was isolated from DIV4 primary hippocampal neurons and tissue samples (forebrain or hindbrain/brainstem) from *Tgf-β2^+/+^* and *Tgf-β2^−/−^* mice at E17.5 using TRIzol reagent (Invitrogen; Darmstadt, Germany) according to manufacturer’s instructions. Subsequently, 1 μg of RNA was reverse-transcribed into cDNA and analyzed with quantitative real-time PCR. Primer sequences used in QRT-PCR are listed in Table 1. PCR conditions were the following: initial denaturation at 95 °C for 10 min, 40 cycles of denaturation at 95 °C for 15 s, annealing at the appropriate temperature of the primer pairs for 30 s, elongation at 72 °C for 30 s and final elongation at 72 °C for 10 min. All reactions were performed in triplicate and the mean Ct value was used to determine gene expression with the 2^−ΔΔCt^ method using *Gapdh* as housekeeping gene.

### 2.5. Chromatin Immunoprecipitation Assay (ChIP)

Chromatin immunoprecipitation assay was performed with an EZ-ChIP kit (Cat# 17-371, Millipore; Darmstadt, Germany) according to manufacturer’s instructions with modifications. Briefly, forebrains from E17.5 *Tgf-β2^+/+^* and *Tgf-β2^−/−^* embryos were dissected and cross-linked in 1% paraformaldehyde for 15 min at room temperature. After homogenization, cell pellets were lysed in SDS lysis buffer and sonicated on ice with 20 cycles of 10 s pulse on/50 s pulse off using a probe sonicator (UW 3100, Bandelin electronic, Berlin, Germany), to obtain 200–1000 bp DNA fragments. Lysates were incubated with anti-AP2β, anti-RNA polymerase II and mouse IgG on a rotating platform overnight at 4 °C. Immunocomplexes were collected with protein G agarose beads, eluates were reverse cross-linked and bound DNA was purified. Immunoprecipitated DNA was analyzed with quantitative real-time PCR using primers specific to the two AP2 binding sequences [20] in mouse *Slc12a5* promoter (Genbank accession number NC_000068.8). Primer sequences are shown in Table 1. PCR conditions were the following: initial denaturation at 95 °C for 10 min, 50 cycles of denaturation at 95 °C for 15 s, annealing at the appropriate temperature of the primer pairs for 30 s, elongation at 72 °C for 30 s and final elongation at 72 °C for 10 min. For the quantification, the adjusted input Ct was calculated by subtracting 6.64 cycles (log2 of 100), as 1% of starting chromatin was used as input. Percent of input for each IP (or negative control IgG) was calculated as follows: 100*2^(Adjusted input Ct- IP Ct)^.

### 2.6. Immunoblotting

Day in vitro (DIV) 4 primary hippocampal neurons, E17.5 forebrain and hindbrain/brainstem tissue or acute brainstem slices were harvested and homogenized, and protein concentration was determined using a Thermo Scientific NanoDrop 2000 spectrophotometer (absorbance at 280 nm). Electrophoresis and blotting procedures were performed as described [32]. Primary antibodies were diluted as follows: KCC2 1:5000–1:10,000, pKCC2 (S940) 1:2000–1:5000, pKCC2 (T1007) 1:2000–1:5000, GAPDH 1:20,000, Na^+^/K^+^-ATPase alpha-1 subunit 1:1000. Blots were developed in enhanced chemiluminescence reagents and signals were visualized on X-ray films. Subsequently, films were scanned and the signal ratio protein of interest/GAPDH for total cell homogenates or KCC2/Na^+^/K^+^-ATPase for surface proteinswas quantified densitometrically. Differences in signal ratio were tested for significance.

### 2.7. Immunocytochemistry

Primary hippocampal neurons were fixed with 4% PFA for 30 min at room temperature. Cells were washed with PBS and treated with 1% SDS for 5 min, blocked with 1% BSA and incubated with primary antibody anti-KCC2 (1:250 in BSA), anti-pKCC2 S940 (1:200 in BSA), anti-pKCC2 T906 (10 μg/mL in BSA) or anti-pKCC2 T1007 (10 μg/mL in BSA) overnight at 4 °C. The incubation with phosphorylation-site-specific sheep antibodies was performed in the presence of 100 μg/mL of the non-phosphorylated form of the phosphorylated peptide used to raise the antibody. Cells were washed with PBS and incubated with donkey anti-rabbit or anti-sheep IgG coupled to Alexa 594 (1:400) for 1 h. Coverslips were washed with PBS and mounted with Fluoromount-G, containing 4′,6′-diamidino-2-phenylindole dihydrochloride (DAPI) (SouthernBiotech, #0100-20) for nuclear staining.

### 2.8. Image Acquisition and Analysis

Images were acquired with a Leica TCS SP8 confocal microscope using an HC PL APO CS2 40×/1.30 or 63×/1.40 oil objective lens, and immunofluorescence intensity was analyzed as described previously [41]. Within each experiment, the confocal microscope settings (laser power, detector gain and amplifier offset) were kept the same for all scans in which the immunofluorescence intensity was compared. Z-stacks of 16–30 optical sections with a step size of 0.4 µm were taken for at least 3 separate fields of view for each experimental condition. Maximum intensity projections were created from the z-stacks. To quantify the protein expression, LAS X software was used to select the area of interest (neuronal soma) and measure the average fluorescence intensity within this area for each cell. Background subtraction was applied to the images.

The values of KCC2 fluorescence intensity within the soma of each cell were used for the classification of neurons with high/low KCC2. The average KCC2 fluorescence intensity of all *wildtype* neurons measured per experiment was set as threshold. For each genotype, neurons exhibiting intensity higher than the threshold were classified as ‘high KCC2’, whereas neurons exhibiting intensity lower than the threshold were classified as ‘low KCC2’. The data are presented as percent of total cells counted per genotype.

### 2.9. Acute Brainstem Slices

*Tgf-β2^+/+^, Tgf-β2^+/−^* and *Tgf-β2^−/−^* embryos at E17.5–18.5 were sacrificed by decapitation, and brains were quickly removed and placed in chilled artificial cerebrospinal fluid (ACSF; 120 mM NaCl, 8 mM KCl, 1.26 mM CaCl_2_, 1.5 mM MgCl_2_, 21 mM NaHCO_3_, 0.58 mM Na_2_HPO_4_, 30 mM glucose). The brainstem was sliced (frontal plane) into 200 µm acute slices using a VT1000S vibratome (Leica). Slices containing the pre-Bötzinger complex were then incubated in the presence or absence of recombinant TGF-β2 (5 ng/mL) for 1h and processed for immunoblotting, immunohistochemistry or surface biotinylation.

### 2.10. Immunofluorescence on Tissue Sections

Mouse embryonic brains or acute brainstem slices were fixed with 4% PFA for 24 h or 2 h, respectively, cryoprotected in 15% sucrose/PBS, embedded in cryomatrix and cryosectioned at 10 µm. Sections were washed three times with PBS-T (PBS + 0.2% Triton-X 100), blocked in 10% normal donkey serum (NDS) in PBS-T for 1 h at room temperature and incubated with primary antibodies anti-KCC2 (1:1500), SST(1:1500) in 10% NDS/ PBS-T for 48 h at 4 °C. Subsequently, sections were washed three times with PBS-T and incubated with secondary antibody donkey anti-rabbit IgG coupled to Alexa-568, donkey anti-goat IgG coupled to Alexa 488 (1:200 in 3% NDS/ PBS-T) and Phalloidin-633 for 1 h at room temperature. Sections were washed three times in PBS-T and mounted with Fluoromount-G with DAPI (#0100-20, SouthernBiotech, Birmingham, USA) for nuclear staining. Images were acquired with a Zeiss Confocal Microscope LSM 510 Meta. Subsequently, SST-positive neurons exhibiting membrane KCC2 were counted in *wildtype* and *Tgf-β2^−/−^* sections. Membrane KCC2 abundance was judged at high magnification and confirmed by line scans (peaks of fluorescence intensity at the periphery of the cells).

### 2.11. Surface Biotinylation of Acute Brainstem Slices

Acute brainstem slices from *wildtype* and *Tgf-β2*-deficient mouse embryos at E18.5 containing the PreBötC were subjected to control or experimental conditions (application of recombinant TGF-β2) and then kept on ice. Isolation of cell surface proteins was performed using the Pierce^®^ (Thermo Fisher Scientific; Dreieich, Germany) cell surface protein isolation kit following the manufacturer’s instructions. Proteins were then processed for immunoblotting with antibodies for KCC2 and Na^+^/K^+^-ATPase, as described above.

### 2.12. Statistical Analysis

Statistical tests were performed as indicated in thetext. All tests were performed in GraphPad Prism, Version 7.04, GraphPad, San Diego, USA for Windows. Data were tested for normal distribution, using a Shapiro–Wilk test and subsequently assessed for homogeneity of variance. If the data passed both tests, further analyses were performed using the two-tailed unpaired Student’s *t*-test. For datasets with unequal variances, Welch’s correction was applied after Student’s *t*-test. Values are reported as mean ± S.E.M., unless otherwise indicated. For datasets with non-normal distributions, the Mann–Whitney rank-sum test was used. For all statistical tests, *p ≤* 0.05 was considered statistically significant, and *p*-values are indicated in the figures as follows: * *p ≤* 0.05, ** *p <* 0.01, *** *p <* 0.001, **** *p* < 0.0001, ^#^ *p* < 0.05 and ^##^ *p* < 0.01.

## 3. Results

### 3.1. KCC2 Expression in Immature Neurons Is Regulated by TGF-β/Activin Signaling

During maturation of most central neurons, the expression of KCC2 is upregulated, resulting in an intracellular Cl^−^ concentration below its electrochemical equilibrium, thereby rendering GABA_A_ responses to hyperpolarizing and inhibitory [1,42]. Early studies have shown that the mechanisms regulating KCC2 expression appear to involve signaling induced by trophic factors [23,24,25,26] rather than GABA_A_-receptor-mediated or ionotropic transmission or neuronal activity [42]. We have previously shown TGF-β-dependent and Rab11b-mediated KCC2 trafficking to the plasma membrane in differentiating and mature neurons [33]. Considering the context-dependent action of TGF-β, we first investigated the effect of TGF-β on KCC2 expression and/or localization in immature neurons.

**Figure 1:**
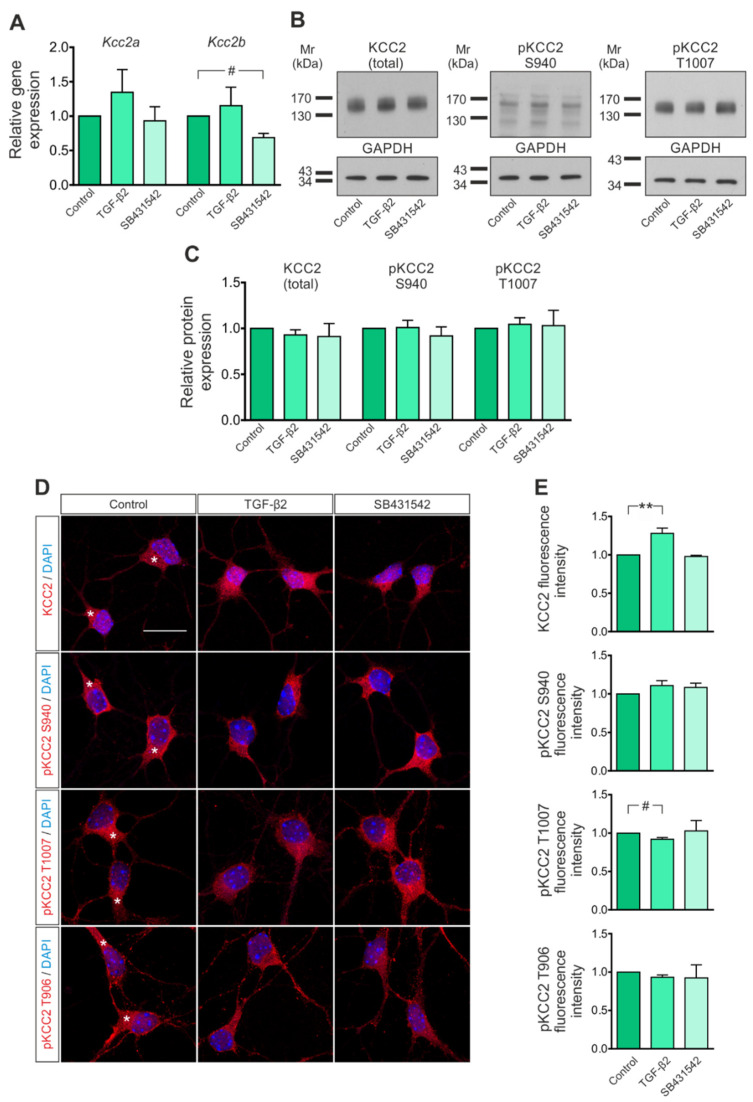
Regulation of *Kcc2* transcript and KCC2 protein in immature mouse hippocampal neurons by TGF-β/activin signaling. (**A**) Quantitative RT-PCR analysis for the splice variants *Kcc2a* and *Kcc2b* on cDNA from primary immature mouse hippocampal neurons treated with either TGF-β2 (2 ng/mL) or 10 µM SB431542 (an ALK4/5/7 inhibitor) for 60 min (^#^ *p* < 0.05 for significant decrease compared to controls, using the two-tailed unpaired Student’s *t*-test with Welch’s correction, *n* = 4–6). (**B**) Representative blots and (**C**) quantification of protein abundance of total and phosphorylated (p)KCC2 at S940 and T1007 by immunoblotting in control immature hippocampal neurons and following treatment with 2 ng/mL TGF-β2 or the ALK4/5/7 inhibitor SB431542 (10 µM); not significant after densitometric analysis of the signal ratio protein of interest: GAPDH and two-tailed unpaired Student’s *t*-test with Welch’s correction, *n* = 5. A total of 20 µg protein was loaded per lane. (**D**) Immunofluorescence confocal microscopy for KCC2 (red) and pKCC2 at S940, T1007 or T906 on mouse primary immature hippocampal neurons. Asterisks indicate intracellular KCC2 localization. Scale bar: 20 µm. (**E**) Quantification of immunofluorescence intensity of total KCC2 and pKCC2 at S940, T1007 and T906 following treatment with TGF-β2 (2 ng/mL) or SB431542 (10 μM) (** *p* < 0.01 for significant increase and ^#^ *p* < 0.05 for significant decrease compared to controls using the two-tailed unpaired Student’s *t*-test with Welch’s correction, *n* = 3–7). All data are shown as mean ± S.E.M. The values of the respective controls were set to 1.

Mouse hippocampal neurons were isolated at E17.5–18.5, cultured for 4 days in vitro, and treated either with 2 ng/mL TGF-β2 or with 10 µM SB431542,—an ALK4/5/7 inhibitor, thus, an inhibitor of TGF-β/activin signaling [35]—for 60 min. Subsequently, transcript expression of the two splice variants of *Kcc2*, *Kcc2a* and *Kcc2b* [18], protein abundance of total and phosphorylated (p)KCC2 as well as subcellular localization were assessed by QRT-PCR, immunoblotting and immunofluorescence microscopy, respectively. Figure 1A shows that exposure of the cultures to exogenous recombinant TGF-β2 had no effect on *Kcc2a* or *Kcc2b* transcript expression (1.35 ± 0.33 fold and 1.15 ± 0.27 fold for *Kcc2a* and *Kcc2b*, respectively, not significant using two-tailed unpaired Student’s *t*-test with Welch’s correction, *n* = 6), whereas treatment with SB431542 significantly downregulated *Kcc2b* (0.69 ± 0.06 fold, ^#^ *p* < 0.05 using two-tailed unpaired Student’s *t*-test with Welch’s correction, *n* = 4) but not *Kcc2a* (0.93 ± 0.20 fold) compared to untreated controls. Immunoblotting for total KCC2 (Figure 1B) and pKCC2 at S940 and T1007 revealed prominent immunoreactive bands at ~135–140 kDa in whole-cell homogenates from controls. Exposure of the cultures to exogenous recombinant TGF-β2 had no effect on total KCC2 or pKCC2 at S940 and T1007 protein expression (Figure 1B,C; 0.93 ± 0.06 fold, 1.01 ± 0.08 fold and 1.04 ± 0.07 fold for total KCC2, pKCC2 at S940 and at T1007, respectively; not significant using two-tailed unpaired Student’s *t*-test with Welch’s correction, *n* = 5) compared to untreated controls. Furthermore, treatment with SB431542 did not affect protein expression of total KCC2 or pKCC2 at S940 and at T1007 (0.91 ± 0.14 fold, 0.92 ± 0.10 fold and 1.03 ± 0.17 fold for total KCC2, pKCC2 at S940 and at T1007, respectively; not significant using two-tailed unpaired Student’s *t*-test with Welch’s correction, *n* = 5) compared to untreated controls.

These data were extended by immunofluorescence confocal microscopy (Figure 1D). Cellular localization of KCC2 in controls was mainly somatic (asterisks), in accordance with previous observations [42]. Quantification of immunofluorescence intensity (Figure 1E) revealed that treatment with TGF-β2 significantly upregulated total KCC2 immunofluorescence (1.28 ± 0.07 fold, ** *p* < 0.01 using two-tailed unpaired Student’s *t*-test with Welch’s correction, *n* = 7), significantly downregulated pKCC2 at T1007 immunofluorescence (0.92 ± 0.02 fold, ^#^ *p* < 0.05 using two-tailed unpaired Student’s *t*-test with Welch’s correction, *n* = 5) and had no effect on pKCC2 at S940 or T906 immunofluorescence (1.11 ± 0.06 fold and 0.93 ± 0.03 fold for pKCC2 at S940 and at T906, respectively; not significant using two-tailed unpaired Student’s *t*-test with Welch’s correction, *n* = 5–6) compared to untreated controls. Treatment with SB431542 had no effect on total KCC2 or phosphorylated KCC2 at S940, T1007 or T906 immunofluorescence (0.98 ± 0.01 fold, 1.08 ± 0.06 fold, 1.03 ± 0.13 and 0.92 ± 0.17 fold for total KCC2 and KCC2 phosphorylated at S940, T1007 and T906 respectively; not significant using two-tailed unpaired Student’s *t*-test with Welch’s correction, *n* = 3–4) compared to untreated controls.

These results suggest that TGF-β/activin signaling is involved in regulation of KCC2 and provide first hints for a contribution of the ligand TGF-β2 in this process.

### 3.2. Expression of Transcription Factors That Potentially Modulate Kcc2 Promoter Activity Is Regulated by TGF-β/Activin Signaling

**Figure 2:**
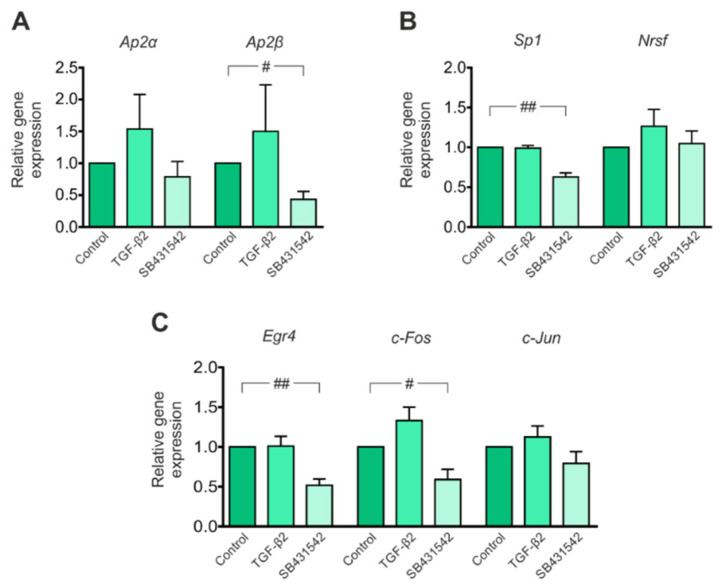
Regulation of expression of transcription factors with putative binding sites on the *Kcc2b* promoter in immature mouse hippocampal neurons by TGF-β/activin signaling. Quantitative RT-PCR analysis for the transcription factors *Ap2α*, *Ap2β*, (**A**) *Sp1*, *Nrsf,* (**B**) *Egr4*, *c-Fos* and *c-Jun* (**C**) on cDNA from primary immature mouse hippocampal neurons treated with either TGF-β2 (2 ng/mL) or 10 µM SB431542 (an ALK4, 5 and 7 inhibitor) for 60 min (^#^ *p* < 0.05 and ^##^ *p* < 0.01 for significant downregulation compared to controls using the two-tailed unpaired Student’s *t*-test with Welch’s correction, *n* = 4–6). All data are shown as mean ± S.E.M. The values of the respective controls were set to 1.

*Kcc2* promoter analysis has uncovered conserved putative binding sites for several transcription factors that may modulate *Kcc2* gene expression, among them two sites for specificity protein 1 (SP1) and activating enhancer binding protein 2 (AP2) and one site for activator protein 1 (AP1), neuron-restrictive silencer element (NRSE) and early growth response 4 (EGR4) [20]. Because these transcription factors have been associated with TGF-β signaling in several cell types [43,44,45], and considering downregulation of *Kcc2b* in immature neurons following exposure to SB431542 (Figure 1A), as a next step, we assessed putative regulation of expression of the transcription factors in mouse primary immature hippocampal neurons treated with either TGF-β2 or with SB431542 for 60 min by QRT-PCR. As illustrated in Figure 2A, exposure of the cultures to exogenous TGF-β2 had no effect on expression of *Ap2α* (1.54 ± 0.54 fold) or *Ap2β* (1.50 ± 0.73 fold), whereas SB431542 significantly downregulated *Ap2β* (0.43 ± 0.12 fold) but not *Ap2α* (0.79 ± 0.24 fold) compared to the untreated controls. A similar regulation pattern was also observed for *Sp1*, *Egr4* and *c-Fos*: treatment of immature hippocampal neurons with exogenous TGF-β2 did not alter expression of *Sp1* (0.99 ± 0.03 fold), *Egr4* (1.01 ± 0.12 fold) or *c-Fos* (1.33 ± 0.17 fold), whereas application of SB431542 significantly downregulated expression of *Sp1* (0.63 ± 0.05 fold), *Egr4* (0.52 ± 0.08 fold) and *c-Fos* (0.59 ± 0.13 fold) compared to the untreated controls. In contrast, expression of *Nrsf* (1.27 ± 0.21 fold and 1.05 ± 0.16 fold for TGF-β2 and SB431542, respectively) and *c-Jun* (1.13 ± 0.14 fold and 0.79 ± 0.15 fold for TGF-β2 and SB431542, respectively) remained comparable for all experimental conditions (^#^ *p* < 0.05 and ^##^ *p* < 0.01 for significant decrease using two-tailed unpaired Student’s *t*-test with Welch’s correction, *n* = 4–6).

These results suggest that TGF-β/activin signaling is involved in regulation of expression of transcription factors with potential to regulate *Kcc2* transcription.

### 3.3. TGF-β2-Dependent Regulation of Kcc2 Gene Expression and KCC2 Phosphorylation State in the Developing Mouse Forebrain

**Figure 3:**
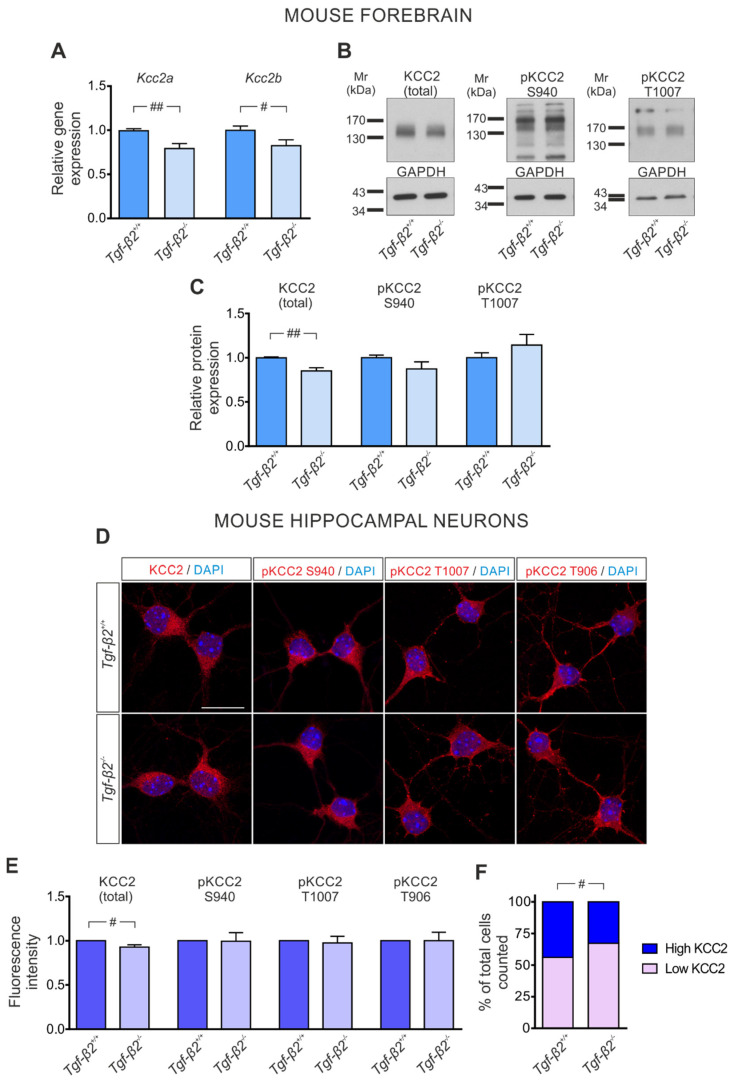
TGF-β2-dependent regulation of KCC2 in the mouse forebrain. (**A**) Quantitative RT-PCR analysis for *Kcc2a* and *Kcc2b* on cDNA from forebrain at embryonic day (**E**) 17.5 of wildtype (*Tgf-β2^+/+^*) and *Tgf-β2^−/−^* mice (^#^ *p* < 0.05 and ^##^ *p* < 0.01 for significant decrease using two-tailed unpaired Student’s *t*-test, *n* = 7 for *Tgf-β2^+/+^* and *n* = 6 for mutant). (**B**) Immunoblot analysis for total KCC2 and for phosphorylated (p)KCC2 at S940 or T1007 in homogenates from forebrain tissue of *Tgf-β2^+/+^* and *Tgf-β2^−/−^* mice. (**C**) Quantification of immunoblots (^##^ *p* < 0.01 for significant decrease after densitometric analysis of the signal ratio protein KCC2: GAPDH and two-tailed unpaired Student’s *t*-test, *n* = 6–7 for wildtype and *n* = 6 for mutant). The data are shown as mean ± S.E.M., relative to the mean of wildtype littermates. The blots are representative for four different litters. A total of 30 µg protein was loaded per lane. (**D**) Cellular localization of KCC2 (red) and phosphorylated (p)KCC2 at S940, T1007 or T906 in primary hippocampal neuronal cultures from *Tgf-β2^+/+^* and *Tgf-β2^−/−^* by immunofluorescence and subsequent confocal microscopy. Scale bar: 20 µm. (**E**) Quantification of labelling intensity (^#^ *p* < 0.05 for significant decrease in *Tgf-β2^−/−^* compared to *wt* using two-tailed unpaired Student’s *t*-test; *~*400 cells were analyzed per genotype for total KCC2 and ~100–200 cells for (p)KCC2 labeling). (**F**) Distribution (%) of counted cells (~400 per genotype) expressing high or low KCC2 between *Tgf-β2^+/+^* and *Tgf-β2^−/−^* (^#^ *p* < 0.05 for significant decrease of high KCC2-expressing cells in *Tgf-β2^−/−^* compared to *Tgf-β2^+/+^*).

Since SB431542 inhibits ALK4/5/7 receptors, several ligands that signal through these receptors are putative candidates for regulating the expression of *Kcc2* transcript (Figure 1A) and/or transcription factors (Figure 2) potent to regulate *Kcc2* transcription. TGF-βs signal through ALK5, and the isoform TGF-β2 has been considered relatively more relevant during neuronal development compared to TGF-β3 [28]. With this background in mind, we investigated a putative relative contribution of the isoform TGF-β2 in regulating *Kcc2* in immature neurons. Therefore, forebrain tissue from wildtype (*Tgf-β2^+/+^*) and *Tgf-β2^−/−^* embryos at E17.5 was isolated and, subsequently, *Kcc2a* and *Kcc2b* transcript expression, KCC2 whole protein and phosphorylation at S940 and T1007 was determined. Figure 3A shows that transcript expression of both *Kcc2a* and *Kcc2b* was significantly downregulated in the forebrain of *Tgf-β2* constitutive null mutants (0.80 ± 0.05 and 0.83 ± 0.06 fold for *Kcc2a* and *Kcc2b*, respectively), compared to that of wildtype (*wt*) littermates (1.00 ± 0.02 and 1.00 ± 0.04 fold for *Kcc2a* and *Kcc2b*, respectively; ^##^ *p* < 0.01 using two-tailed unpaired Student’s *t*-test with Welch’s correction; ^#^ *p* < 0.05 using two-tailed unpaired Student’s *t*-test; *n* = 7 for *wt* and *n* = 6 for mutants). In line with these results, total KCC2 protein in homogenates from forebrain (Figure 3B,C) was significantly downregulated in *Tgf-β2^−/−^* embryos, compared to *wt* littermates (0.85 ± 0.04 vs. 1.00 ± 0.01 fold, ^##^ *p* < 0.01 using two-tailed unpaired Student’s *t*-test with Welch’s correction, *n* = 7 for *wt* and *n* = 6 for mutants). In contrast, phosphorylation state of KCC2 at both S940 (0.87 ± 0.08 vs. 1.00 ± 0.03 fold) and T1007 (1.14 ± 0.12 vs. 1.00 ± 0.05 fold; Figure 3B,C) revealed no significant differences between *wt* and mutants (using two-tailed unpaired Student’s *t*-test with Welch’s correction, *n* = 6–7 for *wt* and *n* = 6 for mutants). The immunoblot data were further confirmed and extended by immunofluorescence for total KCC2 or for pKCC2 at S940, T1007 or T906 (red) on primary hippocampal neurons at DIV4 (Figure 3D). Quantification of labeling intensity (Figure 3E) of total KCC2 showed significant downregulation in *Tgf-β2*-deficient neurons (0.93 ± 0.03 fold) (^#^ *p* < 0.05 for significant decrease using two-tailed unpaired Student’s *t*-test) compared to *wt*, whereas pKCC2 at S940, T1007 and T906 labeling intensity was comparable between *wt* and *Tgf-β2*-deficient (0.99 ± 0.10 fold, 0.97 ± 0.07 fold and 1.00 ± 0.09 fold for S940, T1007 and T906, respectively; *n* = 3–4) hippocampal neurons, thus matching the immunoblot data. Furthermore, as shown in Figure 3F, the proportion of cells exhibiting high KCC2 abundance (as described in Materials and Methods, 2.8) was significantly decreased in *Tgf-β2^−/−^* (32.78 ± 3.34%) compared to *wt* (43.91 ± 1.36%). Subsequently, the proportion of cells classified as expressing low KCC2 was significantly increased in *Tgf-β2^−/−^* (67.22 ± 3.34%) compared to *wt* (56.09 ± 1.36 %) (^#^ *p* < 0.05 for significant decrease using two-tailed unpaired Student’s *t*-test with Welch’s correction, *n* = 7 for *wt* and *n* = 8 for mutants). Moreover, similar to the results obtained in *wt*, KCC2 cellular localization was found to be exclusively intracellular, matching the fact that KCC2 in DIV4 hippocampal neurons is not yet functional.

### 3.4. Transcriptional Regulation of Kcc2 via TGF-β2

**Figure 4:**
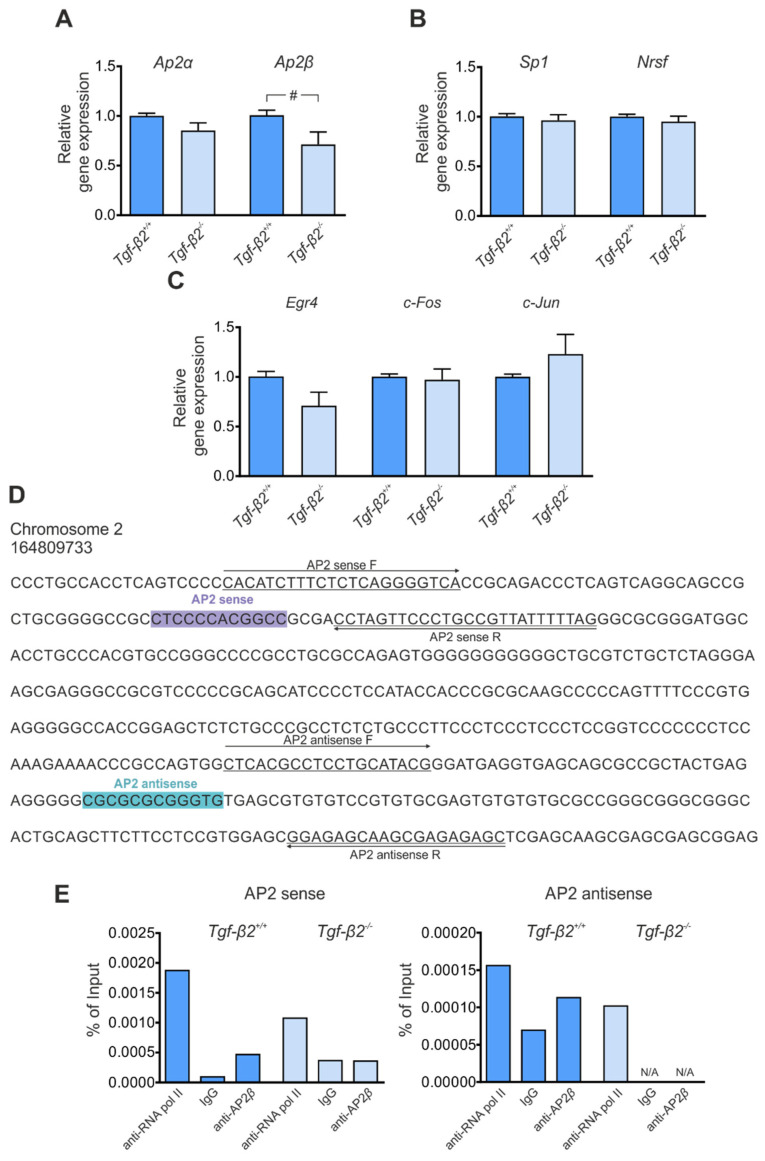
Transcriptional regulation of *Kcc2* by TGF-β2 in mouse forebrain. Quantitative RT-PCR analysis for the transcription factors *Ap2α*, *Ap2β*, (**A**) *Sp1*, *Nrsf,* (**B**) *Egr4*, *c-Fos* and *c-Jun* (**C**) on cDNA from mouse forebrain at embryonic day (**E**) 17.5 from *Tgf-β2^+/+^* and *Tgf-β2^−/−^* mice (^#^ *p* < 0.05 for significant downregulation compared to *wildtype* using two-tailed unpaired Student’s *t*-test, *n* = 6–7). Data are shown as mean ± S.E.M. relative to mean of *wildtype* littermates. (**D**) AP2 sense (purple) and antisense (turquoise) binding sites to the *Kcc2* promoter. Underlined sequences represent forward (F) and reverse (R) primer sequences used to detect AP2 binding. (**E**) Quantitative PCR for putative AP2 sense and antisense sequences on *Kcc2* promoter, following chromatin immunoprecipitation assay with AP2β antibody in *Tgf-β2^+/+^* and *Tgf-β2^−/−^* mouse forebrain. RNA polymerase II and mouse IgG represent positive and negative control, respectively. The quantification is representative of *n* = 4 per genotype.

Having shown that expression of transcription factors with putative binding sites to *Kcc2b* promoter are regulated following inhibition of TGF-β/Activin signaling pathway in DIV4 hippocampal neurons (Figure 2), together with the observation of reduced *Kcc2a* and *Kcc2b* transcript in forebrain of *Tgf-β2* deficient mice (Figure 3A), we tested the hypothesis that TGF-β2 regulates *Kcc2* expression via non-canonical signaling. Therefore, first, we investigated a putative contribution of the ligand TGF-β2 on regulating transcript expression of the relevant transcription factors *Ap2α*, *Ap2β*, *Sp1*, *Nrsf*, *Egr4*, *c-Fos* and *c-Jun*. The results are shown in Figure 4A–C. Interestingly, in contrast to the results obtained in Figure 2, in *Tgf-β2* mutants, only the expression of *Ap2β* was significantly downregulated compared to *wt* (Figure 4A, 0.71 ± 0.12 vs. 1.01 ± 0.05 fold; ^#^ *p* < 0.05 using two-tailed unpaired Student’s *t*-test, *n* = 7 for *wt* and *n* = 6 for mutants), whereas expression of *Ap2α*, *Sp1*, *Nrsf*, *Egr4*, *c-Fos* and *c-Jun* was comparable between the genotypes (Figure 4B,C; 0.85 ± 0.08 vs. 1.00 ± 0.03 fold, 0.96 ± 0.06 vs. 1.00 ± 0.03 fold, 0.95 ± 0.05 vs. 1.00 ± 0.02 fold, 0.71 ± 0.14 vs. 1.00 ± 0.05 fold, 0.97 ± 0.11 vs. 1.00 ± 0.03 fold and 1.23 ± 0.20 vs. 1.00 ± 0.03 fold for *Ap2α*, *Sp1*, *Nrsf*, *Egr4*, *c-Fos* and *c-Jun* respectively; not significant using two-tailed unpaired Student’s *t*-test, *n* = 7 for *wt* and *n* = 6 for mutants). Subsequently, to provide conclusive evidence of TGF-β2-mediated endogenous AP2β binding to the *Kcc2* promoter, we performed ChIP in forebrain tissue of *wt* and *Tgf-β2*-deficient E17.5 embryos (Figure 4D,E). AP2β was immunoprecipitated using an antibody against AP2β, and qPCR was subsequently performed to amplify DNA fragments bound to AP2β. Therefore, primers flanking the AP2 sense and antisense binding sequences on *Kcc2* promoter were used, as shown in Figure 4D. Figure 4E shows a representative quantification for AP2β binding. AP2β binding signal in AP2 sense sequence of *Kcc2* promoter was detected in *Tgf-β2^+/+^* (0.00047% of input) whereas IgG binding signal was almost absent (0.00009% of input). In contrast in *Tgf-β2^−/−^*, the binding signal of AP2β (0.00036% of input) was comparable with IgG (0.00036% of input), demonstrating that AP2β binding was absent in mutants. Similar results were also obtained for the AP2 antisense sequence. In *Tgf-β2^+/+^*, AP2β binding signal was observed (0.00011% of input) whereas in *Tgf-β2^−/−^*, AP2β binding signal was undetectable. RNA polymerase II served as positive control (0.00187 and 0.00107% of input for *Tgf-β2^+/+^* and *Tgf-β2^−/−^*, respectively, for AP2 sense and 0.00016 and 0.00010% of input for *Tgf-β2^+/+^* and *Tgf-β2^−/−^*, respectively, for AP2 antisense). These results provide a first hint that TGF-β2 might regulate *Kcc2* transcription acting upstream of AP2β.

### 3.5. Loss of TGF-β2 Prevents Developmental Dephosphorylation of KCC2 at T1007

**Figure 5:**
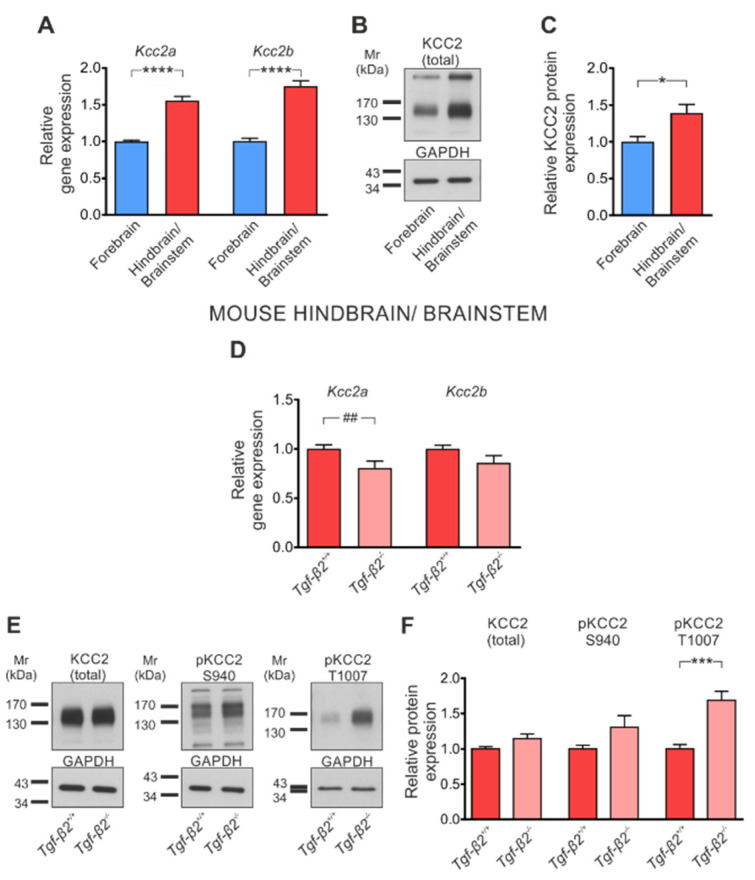
TGF-β2-dependent regulation of KCC2 in the mouse hindbrain/brainstem. (**A**) Quantitative RT-PCR analysis for *Kcc2a* and *Kcc2b* on cDNA from either mouse forebrain or hindbrain/brainstem at embryonic (E) day 17.5 (**** *p* < 0.0001 for significant upregulation compared to forebrain using two-tailed unpaired Student’s *t*-test with Welch’s correction, *n* = 10). (**B**) Immunoblot analysis for total KCC2 in tissue homogenates from mouse forebrain and hindbrain/brainstem at E17.5. (**C**) * *p* < 0.05 for significant increase after densitometric analysis of the signal ratio KCC2: GAPDH and Student’s *t*-test, *n* = 6. Data are shown as mean ± S.E.M. relative to the mean of forebrains. The blots are representative of six different litters. A total of 30 µg protein was loaded per lane. (**D**) Quantitative RT-PCR analysis for *Kcc2a* and *Kcc2b* on cDNA from hindbrain/brainstem at embryonic day (E) 17.5 of *Tgf-β2^+/+^* and *Tgf-β2 ^−/−^* mice (^##^ *p* < 0.01 using Mann–Whitney test, *n* = 7 for *wildtype* and *n* = 6 for mutant). (**E**) Immunoblot analysis for total KCC2 and for phosphorylated (p)KCC2 at S940 or T1007 in tissue homogenates from hindbrain/brainstem of *Tgf-β2^+/+^* and *Tgf-β2^−/−^* mice. (**F**) *** *p* < 0.001 for significant increase after densitometric analysis of the signal ratio pT1007: GAPDH and two-tailed unpaired Student’s *t*-test, *n* = 6 for *wildtype* and *n* = 6 for mutant. The blots are representative for four different litters. A total of 30 µg protein was loaded per lane. Data are shown as mean ± S.E.M. relative to the mean of *wildtype* littermates.

KCC2 expression follows the pattern of brain structure development. Given that the brainstem matures earlier than the forebrain during CNS development, we tested the hypothesis that the effect of TGF-β2 on KCC2 might be different in the hindbrain/brainstem. Therefore, we determined *Kcc2* transcript expression, protein abundance and phosphorylation state in hindbrain/brainstem tissue of *wt* and *Tgf-β2*-deficient mice. Figure 5A illustrates that in *wt* E17.5 embryos, expression of both *Kcc2a* (1.56 ± 0.06 fold) and *Kcc2b* (1.75 ± 0.07 fold) was significantly upregulated in the hindbrain/brainstem compared to the forebrain (1.00 ± 0.02 and 1.00 ± 0.04 for *Kcc2a* and *Kcc2b*, respectively), as assessed by QRT-PCR (**** *p* < 0.0001 using two-tailed unpaired Student’s *t*-test with Welch’s correction, *n* = 10), matching previous observations [18]. In accordance with these results, total KCC2 protein at ~140 kDa (Figure 5B,C) was significantly upregulated in hindbrain/brainstem (1.39 ± 0.12 fold), compared to the respective forebrain (1.00 ± 0.07) (* *p* < 0.05 using two-tailed unpaired Student’s *t*-test, *n* = 6). These data confirm the view that the brainstem matures earlier compared to the forebrain and serve as a proof of concept of our experimental design. In the hindbrain/brainstem of *Tgf-β2^−/−^* mice (Figure 5D), transcript expression of *Kcc2a* (0.80 ± 0.07 fold) but not *Kcc2b* (0.86 ± 0.07 fold) was significantly downregulated compared to their *wt* littermates (1.00 ± 0.04 fold and 1.00 ± 0.03 fold for *Kcc2a* and *Kcc2b*, respectively; ^##^ *p* < 0.01 using Mann–Whitney test, *n* = 7 for *wt* and *n* = 6 for *Tgf-β2^−/−^*). In contrast, total KCC2 protein abundance was comparable in the hindbrain/brainstem of *wt* and *Tgf-β2^−/−^* as determined by Western blot (Figure 5E). Indeed, quantification (Figure 5F) revealed no significant differences in band intensity (1.00 ± 0.03 and 1.15 ± 0.06 fold for *wt* and *Tgf-β2^−/−^*, respectively). Similarly, no significant difference on pS940 was observed between *Tgf-β2^−/−^* and *wt* littermates (Figure 5E,F; 1.30 ± 0.16 and 1.00 ± 0.05 fold for *Tgf-β2^−/−^* and *wt,* respectively). Interestingly, in *Tgf-β2^−/−^* hindbrain/brainstem, phosphorylation of KCC2 at T1007 (Figure 5E,F) was significantly upregulated (1.69 ± 0.13 fold) compared to the *wt* littermates (1.00 ± 0.06 fold; *** *p* < 0.001 using two-tailed unpaired Student’s *t*-test, *n* = 6).

These results demonstrate TGF-β2-dependent regulation of KCC2 T1007 phosphorylation. Since T1007 phosphorylation has inhibitory effects on KCC2 activity, these results implicate putative TGF-β2-dependent regulation of KCC2 activity through modulating T1007 phosphorylation state.

### 3.6. Impaired KCC2 Membrane Expression in Pre-Bötzinger Complex of Tgf-β2^−/−^ Mice

Based on the results in the hindbrain/brainstem showing that KCC2 phosphorylation at T1007 is considerably increased in *Tgf-β2^−/−^* (Figure 5E,F) together with previous reports showing that mice expressing homozygous phosphomimetic KCC2 mutations at T906/T1007 die early postnatally due to impaired rhythmogenesis in respiratory networks [14,15]—a phenotype that is similar to *Tgf-β2^−/−^* mutants [34]—we examined the KCC2 phenotype in a distinct region of the ventrolateral brainstem that is considered to be the site for respiratory rhythm generation, namely the pre-Bötzinger complex (preBötC) [46]. This nucleus is one of the first to develop a functional neuronal network requiring GABAergic KCC2-mediated inhibition [47,48]. Moreover, we have previously reported that *Tgf-β2^−/−^* mice show impaired synaptic neurotransmission, implying GABAergic and/or glutamatergic mechanisms [49]. Here, we tested the hypothesis that KCC2 membrane abundance may be impaired in preBötC neurons of *Tgf-β2^−/−^* mutants.

A subset of neurons within the preBötC co-express the neurokinin 1 receptor (NK1R) and somatostatin (SST) [50]. Phox2b is expressed in the parafacial respiratory group within the ventrolateral medulla [51] and, hence, serves as a marker to distinguish the neurons of the preBötC from neighboring neuronal subpopulations (Figure S1) in tissue sections from fixed mouse brains at E18.5.

**Figure 6:**
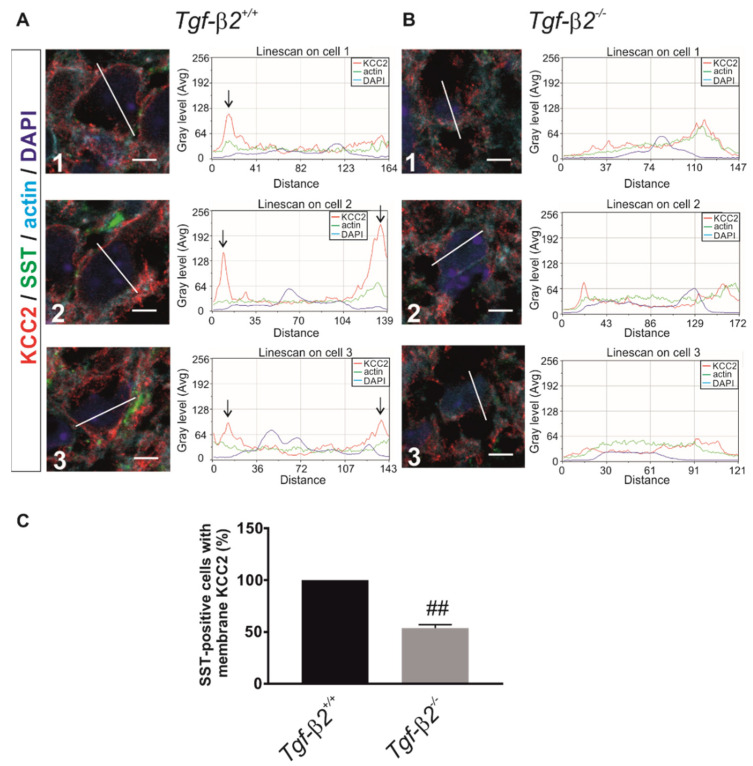
Membrane KCC2 in neurons of the pre-Bötzinger complex is reduced in *Tgf-β2*^−/−^ mice. Fixed brainstem cryosections from *Tgf-β2^+/+^* (**A**) and *Tgf-β2^−/−^* (**B**) embryos at E18.5 immunolabeled with antibodies against KCC2 (red), somatostatin (SST; green) and phalloidin (actin; turquoise). Nuclei were labeled with DAPI (deep blue). KCC2 distribution profile as visualized by line scans for KCC2 (red line), actin (green line) and DAPI (blue line) immunofluorescence for 3 randomly selected neurons in *Tgf-β2^+/+^* (A1–A3) and *Tgf-β2^−/−^* (B1–B3). Scale bars: 5 µm. (**C**) The number of SST-positive neurons showing KCC2 immunofluorescence at the plasma membrane in *Tgf-β2^+/+^* and *Tgf-β2^−/−^* mutants by analysis of approximately 600 neurons. Counted SST-positive preBötC neurons with membrane KCC2 in *Tgf-β2^+/+^* was set to 100%. ^##^ *p* < 0.01 for significant decrease in *Tgf-β2^−/−^* compared to *Tgf-β2^+/+^* (two-tailed unpaired Student’s *t* test with Welch’s correction, *n* = 3 animals per genotype).

In fixed brainstem tissue sections from *wt* mouse embryos, KCC2 immunoreactivity is located in the periphery of preBötC neurons. Figure 6(A1–A3) shows line scans for KCC2 (red), actin (green) and DAPI (blue) for three randomly depicted neurons (numbered 1, 2 and 3). Peaks for KCC2 immunolabeling (arrows) are present at the periphery of neuronal cell bodies, suggesting labeling of the plasma membrane. In contrast, representative line scans for three neurons from the *Tgf-β2^−/−^* mice (Figure 6(B1–B3)) reveal a predominantly intracellular distribution of KCC2 immunoreactivity. We further quantified the KCC2 distribution pattern in somatostatin-positive preBötC neurons by analyzing ~600 neurons/animal/genotype (Figure 6C) and revealed that in *Tgf-β2^−/−^* mice at E18.5, the number of cells exhibiting membrane KCC2 was significantly reduced (53.76 ± 3.28%; ^##^ *p* < 0.01 using two-tailed unpaired Student’s *t*-test with Welch’s correction, *n* = 3) compared to *wildtype* littermates. 

These results demonstrate reduced KCC2 membrane abundance in the preBötC of *Tgf-β2^−/−^* mutants and implicate that this phenotype might contribute to the respiratory failure observed in *Tgf-β2^−/−^* mice [34].

### 3.7. Rescue of KCC2 Phenotype in Tgf-β2^−/−^ Neurons by Exogenous TGF-β2

**Figure 7:**
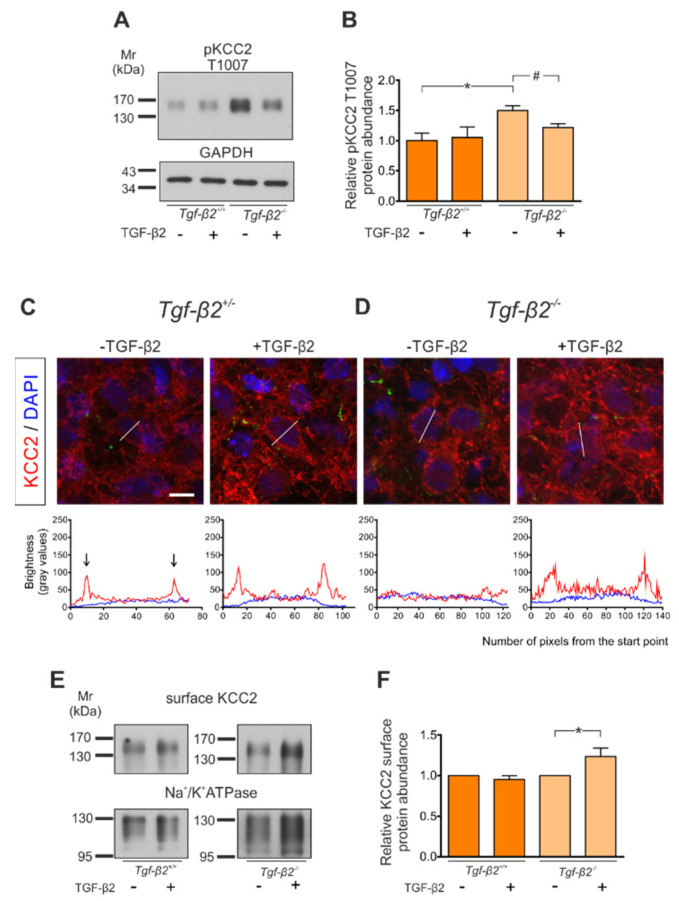
Application of exogenous recombinant TGF-β2 regulates phosphorylation and membrane expression of KCC2 in brainstem of *Tgf-β2^−/−^* mutants. Acute slices from preBötC complex of E17.5–E18.5 from wildtype (*Tgf-β2^+/+^ or Tgf-β2^+/−^*) and *Tgf-β2^−/−^* mutants were cultured in the presence or absence of recombinant human TGF-β2 (5 ng/mL) for 60 min. (**A**,**B**) Immunoblot analysis for phosphorylated (p)KCC2 at T1007 in homogenates from preBötC brainstem slices of *Tgf-β2^+/+^* and *Tgf-β2^−/−^* mutant mice (**B**); * *p* < 0.05 for significant increase and ^#^ *p* < 0.05 for significant decrease after densitometric analysis of the signal ratio pT1007: GAPDH and two-tailed unpaired Student’s *t*-test, *n* = 3 for *Tgf-β2^+/+^* and *n* = 4 for mutants. A total of 10 µg protein were loaded per lane. Data are shown as mean ± S.E.M. relative to the mean of untreated *wt* controls. (**C**,**D**) Fixed cryosections immunolabeled for KCC2 (red). Nuclei were labeled with DAPI (blue). Representative line scans (out of three animals/genotype) for KCC2 (red) and DAPI (blue) illustrate KCC2 labeling pattern from randomly selected neurons for each experimental condition. Scale bar: 30 µm. (**E**,**F**) Immunoblot analysis following biotinylation of surface proteins of acute brainstem slices containing the preBötC from *Tgf-β2^+/+^* and *Tgf-β2^−/−^* mutant mice in the presence or absence of exogenous TGF-β2 (* *p* ≤ 0.05 for significant increase after densitometric analysis of the signal ratio KCC2: Na^+^/K^+^-ATPase and two-tailed unpaired Student’s *t*-test, *n* = 5/genotype; 50 µg protein was loaded per lane). The data are shown as mean ± S.E.M., relative to the untreated controls.

Impaired developmental dephosphorylation of KCC2 at T1007 in *Tgf-β2^−/−^* hindbrain/brainstems can be partly rescued by exogenous TGF-β2, as illustrated in Figure 7A,B. Acute brainstem slices containing the preBötC from *wt* and *Tgf-β2* mutant embryos at E17.5 were exposed to exogenous TGF-β2 (5 ng/mL) for 60 min and T1007 phosphorylation was subsequently determined by immunoblotting. The results show that when *wt* slices were exposed to exogenous TGF-β2, pKCC2 T1007 was comparable (1.05 ± 0.17 fold) to the untreated *wt* controls (1.00 ± 0.13 fold). Interestingly, in untreated *Tgf-β*2^−/−^ brainstem slices, pKCC2 T1007 was significantly upregulated (1.50 ± 0.08 fold) compared to *wt*, an effect that was rescued (1.22 ± 0.06 fold) following exposure of the slices to exogenous TGF-β2 for 60 min at the levels of the treated *Tgf-β2^+/+^* slices (* *p* < 0.05 and *^#^ p* < 0.05 for significant upregulation and downregulation, respectively, using two-tailed unpaired Student’s *t*-test; *n* = 3 for *wt*, *n* = 4 for mutants).

Notably, WNK1-mediated inhibitory phosphorylation of KCC2 at T1007 regulates KCC2 activity by altering its surface expression [11]. Therefore, we next assessed whether TGF-β2 treatment can rescue the *Tgf-β2^−/−^* phenotype (Figure 6). We used acute brainstem slice cultures containing the preBötC from *Tgf-β2^−/−^* at E18.5 and cultured them in the presence or absence of exogenous TGF-β2 for 60 min. Subsequently, slices were fixed and processed for KCC2 immunofluorescence. Line scans from randomly depicted neurons are shown in Figure 7C,D. In *Tgf-β2^+/−^* acute brainstem slices, KCC2 immunolocalization (Figure 7C) was similar to *wt* (Figure 6A), showing distinct peaks of line scans at the cell periphery (arrows). In contrast, consistent with the in vivo analysis (Figure 6B), line scans in *Tgf-β2^−/−^* acute brainstem slices revealed a predominantly intracellular localization of KCC2 immunoreactivity (Figure 7D). Treatment of *Tgf-β2^−/−^* acute slices with exogenous TGF-β2 resulted in a shift of KCC2 immunoreactivity to the cell periphery, as illustrated in the respective line scan (Figure 7D). Figure 7C also reveals that treatment of *Tgf-β2^+/−^* slices with TGF-β2 did not modify the localization pattern of KCC2 immunoreactivity.

Incorporation of KCC2 to the membrane in *Tgf-β2^−/−^* following TGF-β2 treatment of acute brainstem slices was further determined by surface biotinylation experiments. As shown in Figure 7Ε,F, biotinylation of cell surface proteins showed a significant increase for the KCC2 ~135kDa band in *Tgf-β*2^−/−^ acute slices, treated with exogenous recombinant TGF-β2 for 60 min (1.24 ± 0.1 fold; * *p* ≤ 0.05 using two-tailed unpaired Student’s *t*-test, n = 5), compared to the untreated slices. In contrast, KCC2 surface expression was not altered in *wt* acute slices following application of TGF-β2 (0.95 ± 0.05 fold).

These data highlight the biological significance of TGF-β2 for the subcellular distribution of KCC2 in vivo.

## 4. Discussion

The molecular mechanisms responsible for KCC2 regulation during maturation of neuronal networks are not fully elucidated. In the present study, we investigated the impact of TGF-β2 on regulating KCC2 in neurons at different developmental stages. We used primary immature neurons from mouse hippocampi at E17.5 and mouse forebrain and hindbrain/brainstem tissue at E17.5 from TGF-β2 mutants. The advantage, thereby, is that at this developmental stage, these brain areas reveal distinct timetables for neurogenesis. Whereas brainstem neurons show the earliest maturation, in the forebrain, neuronal development is largely delayed.

Our results show multiple and divergent roles of action of TGF-β2 on KCC2 during neuronal maturation. The main novel findings are: (1) TGF-β2 positively regulates KCC2 transcription in immature neurons, possibly via binding of AP2β in the *Kcc2* promoter; (2) TGF-β2 contributes to the developmental regulation of the inhibitory phosphorylation at T1007; and (3) TGF-β2 regulates membrane expression of KCC2 in the differentiated neurons of the preBötC. 

### 4.1. TGF-β2-Dependent Regulation of KCC2 Transcription in Immature Neurons

The results show that in immature mouse hippocampal neurons, a time point when KCC2 is not yet functional [52,53], inhibition of the ALK receptors 4/5/7 significantly downregulates *Kcc2b* transcript, whereas in gain-of-function experiments—by exposing the immature neurons to exogenous TGF-β2—no effect on *Kcc2* transcript expression was detected (Figure 1A). The latter observation can be explained by the presence of endogenously expressed TGF-β2. Indeed, endogenous TGF-β2 may saturate signaling so that the application of exogenous TGF-β2 has no further effects while the receptor inhibitor does. However, in gain-of-function experiments, KCC2 immunofluorescence is significantly upregulated. With regard to KCC2 whole-protein and pKCC2 T1007 abundance following application of exogenous TGF-β2, there is an inconsistency between the immunoblot data (Figure 1C) that show no protein expression changes and the immunofluorescence data (Figure 1E) that suggest altered protein expression levels. However, it is reasonable to assume that there may well be differences within the cellular populations in the hippocampus that give rise to differences between gross protein expression levels and immunofluorescence in single cells. Moreover, quantification of KCC2 immunofluorescence intensity was performed exclusively in the neuronal soma and not in the dendrites; therefore, the effect could be “diluted” in the whole-protein samples used for Western blot analysis. In forebrain tissue of E17.5 *Tgf-β2* mutants, KCC2 expression was found significantly decreased at both the transcript and protein level, and in primary hippocampal neuronal cultures, KCC2 immunofluorescence intensity was significantly decreased as well, accompanied by a reduced number of hippocampal neurons expressing high KCC2 abundance (Figure 3), suggesting that TGF-β2 may contribute to the generation of an initial KCC2 vesicular pool that can potentially be recruited at later stages of development. Consistent with this view, the KCC2 protein was present in the somata and dendrites of immature neurons in an intracellular distribution manner rather than plasma membrane-bound. The nature of “high KCC2”- and “low KCC2”-expressing neurons is not clear; however, these experiments were performed in mouse primary hippocampal cultures, containing a mixture of neurons at different (not synchronized) developmental stages. Our results also demonstrate that the effects observed after pharmacological blocking of the TGF-β/activin pathway can be at least partly attributed to TGF-β2. It should be noted, however, that other ligands that signal through ALK4/5/7 may regulate *Kcc2* transcript as well. With regard to other TGF-β isoforms, TGF-β3,—an isoform expressed in the cells as well—cannot compensate for the loss of TGF-β2, demonstrating that the observed actions are isoform-specific. These results extend the molecular network responsible for triggering—and thereafter, progressively upregulating—KCC2 gene expression during development by introducing TGF-β2 as a novel molecular determinant and highlighting a growth-factor-dependent increase in *Kcc2* transcription.

Indeed, to date, only neurotrophins, namely BDNF and neurturin, are identified as inducing significant ERK1/2-dependent upregulation of the *Egr4* transcription factor and increasing *Kcc2* mRNA and protein levels in immature cultured hippocampal neurons [23,24]. However, the effects of BDNF in immature neurons are controversial. Increased *Kcc2* mRNA levels were found in embryos overexpressing BDNF [54], and KCC2 expression was decreased in early postnatal TrkB-deficient mice [55]. On the other side, surprisingly, *Bdnf^−/−^* mice do not exhibit any phenotype for either developmental upregulation or function of KCC2 [56]. Besides EGR4, the transcription factors USF1 and USF2 activate [19], whereas REST represses *Kcc2* transcription [21,22]. Here, we show that expression of the transcription factors *Ap2β*, *Sp1*, *Egr4* and *cFos* was significantly downregulated in immature neurons following blocking of the TGF-β/activin pathway (Figure 2), but only *Ap2β* expression was downregulated in forebrains from *Tgf-β2* null mice. Subsequent ChIP assay revealed that the transcription factor AP2β binds to the *Kcc2* promoter. Notably, this binding was absent in *Tgf-β2* mutants, implicating that a TGF-β2-dependent increase in *Kcc2* transcription may be mediated via the non-canonical pathway and subsequent binding of AP2β to the *Kcc2* promoter. Further functional studies are needed to provide conclusive evidence for activation of *Kcc2* promoter through TGF-β-mediated AP2β binding. Moreover, the observation that inhibition of the ALK4/5/7 pathway downregulated several transcription factors with putative binding sites on *Kcc2* promoter implies that other ligands may regulate *Kcc2* transcript by unknown TGF-β-independent-signaling as well. Both BDNF and TGF-β2 exert different actions relative to KCC2 in immature and differentiating/mature neurons. BDNF downregulates KCC2 transcript and protein in an activity-dependent manner [25,26], whereas TGF-β2 has an impact on KCC2 posttranslational modifications, as will be discussed below.

### 4.2. TGF-β2-Dependent Regulation of KCC2 Phosphorylation at T1007

A further novel finding of the present work is the TGF-β2-dependent regulation of KCC2 phosphorylation at T1007. The following lines of experimental evidence support this view: (1) exposure of immature neurons to exogenous TGF-β2 significantly reduced T1007 phosphorylation (Figure 1E); (2) in hindbrain/brainstem tissue of *Tgf-β2*-deficient mice T1007 phosphorylation was significantly increased (Figure 5F); and (3) increased T1007 phosphorylation in acute brainstem slices containing the preBötC in the *Tgf-β2* mutants was prevented following treatment with exogenous TGF-β2 (Figure 7B). To our knowledge, this is the first demonstration of growth-factor-dependent regulation of post-translational modification of KCC2. It is unequivocally demonstrated that alterations in KCC2 phosphorylation contribute in regulating functional KCC2 expression, and even the developmental upregulation of KCC2 activity is at least partially mediated through (de)phosphorylation events. However, it has also been appreciated that phosphoregulation of KCC2 is complex, and likely more than one phosphosite contributes in stimulus-induced activation or inhibition of KCC2, as reflected in the observation that staurosporine, a broad kinase inhibitor, increases KCC2 activity in immature neurons [12]. T906/T1007 phosphorylation is decreasing in the course of neuronal maturation, is mediated by the WNK-SPAK/OSR1 cascade and inversely correlates with KCC2 function [11,12,13]. Along this line, in mice with constitutive dephosphorylation of T906/T1007, the onset of seizures is delayed and the severity of seizures attenuated [16]. Our results demonstrate increased T1007 phosphorylation in hindbrains of *Tgf-β2* mutants, suggesting that TGF-β2 signaling, either by activation of a phosphatase and/or inhibition of WNK1-SPAK/OSR1 or other kinase, regulates (de)phosphorylation of KCC2 at T1007. The exact signaling cascade or cross-talk between pathways needs to be elucidated.

In contrast, PKC-mediated phosphorylation at S940 enhances KCC2 function [10], and in mice harboring the S940 mutation, the development and lethality of status epilepticus was accelerated [17]. Our results show that phosphorylation of KCC2 at S940 is neither regulated by TGF-β2 in immature (Figure 1B–E and Figure 3B–E) nor mature (Figure 5F) neurons. Taken into consideration the numerous putative KCC2 phosphosites and the several non-canonical TGF-β pathways, it is likely that additional phosphosites may be regulated by TGF-β2, thereby contributing to dynamic activating and inhibitory (de)phosphorylation events that are required for fine tuning of KCC2 transport capacity. Since the necessary tools are, therefore, lacking, a phosphoproteomic approach in *wt* and *Tgf-β2* mutants could be a way forward. 

Phosphorylation-induced transport of KCC2 to the cell membrane versus intrinsic properties, by means of transport activity, has been controversial. In mice harboring S940 mutation, surface expression of KCC2 was comparable between wild type and mutants [17], another study however, showed decreased rate of KCC2 internalization from the plasma membrane due to PKC-dependent phosphorylation at S940 [10]. Mice with constitutive dephosphorylation T906A/T1007A reveal no difference on KCC2 surface expression [16], whereas T906 and T1007 phosphorylation was shown to regulate KCC2 activity by altering its surface abundance [11]. We tested the hypothesis that increased T1007 phosphorylation in hindbrain/brainstem of *Tgf-β2* mutants is linked to decreased membrane KCC2 expression. 

### 4.3. TGF-β2-Dependent Regulation of Membrane KCC2 in the PreBötC

In the context of the present work it is important that in vivo, mice expressing homozygous phosphomimetic KCC2 mutations at T906/T1007 die early postnatally due to impaired rhythmogenesis in respiratory networks [14,15]. Strikingly, *Tgf-β2^−/−^* and *Kcc2^−/−^* mice reveal similar phenotypes, exhibiting impaired respiratory rhythmogenesis, and both mutations are lethal immediately after birth due to respiratory failure [34,47]. The preBötC neurons in the brainstem are responsible for respiratory rhythmogenesis [48] and show the earliest neuronal maturation. Here, we show that in *Tgf-β2^−/−^* mutants about half of preBötC neurons lack membrane KCC2 compared to the *wildtype* littermates (Figure 6), an observation matching increased T1007 phosphorylation in brainstem slices containing the preBötC (Figure 7A,B). Interestingly, exogenous TGF-β2 rescued the increased T1007 phosphorylation and increased membrane KCC2 in brainstem slices containing the preBötC in *Tgf-β2* mutants (Figure 7C–F) as determined by immunofluorescence and following biotinylation of surface proteins. In a previous study, we have also shown that TGF-β signaling regulates trafficking of KCC2 to the neuronal membrane in differentiating and mature neurons via Rab11b [33]. Taken into consideration that phosphorylation events have potential to rapidly modulate KCC2, we suggest that TGF-β2 is able to regulate KCC2 by several modes of action that may occur at the same time or sequentially in a context-dependent manner. We can even speculate that deficits in membrane-bound KCC2 alone—either through T1007 phosphorylation-dependent reduced membrane KCC2 abundance in preBötC neurons and/or impaired Rab11b-mediated KCC2 trafficking, or in conjunction with other synaptic impairments—could contribute to the respiratory failure associated with the *Tgf-β2^−/−^* phenotype [49]. 

In summary, the present work has provided new mechanistic insights for TGF-β2-mediated regulation of KCC2 gene expression, posttranslational modification and membrane expression. We therefore propose TGF-β2 as a major regulator of KCC2 with putative implications during pathophysiological conditions.

## Figures and Tables

**Table 1:** List of primers used for quantitative RT-PCR and ChIP-qPCR.

**Primers Used for QRT-PCR**			
**Primer Name**	**Sequence**	**GenBank** **Accession No.**	**Source**
*Ap2α* F	GCGGCCCAATCCTATCCT	BC007471	[36]
*Ap2α* R	CCATGGGAGATGAGGTTGAAG		
*Ap2β* F	AAAGCTGTCTCACGCACTTCAGT	NM_009334	
*Ap2β* R	AGCGCAGCGCAAATGG		
*c-Fos* F	CCAGTCCTCACCTCTTCCAG	NM_010234.3	[37]
*c-Fos* R	TCCAGCACCAGGTTAATTCC		
*c-Jun* F	AAAACCTTGAAAGCGCAAAA	NM_010591.2	
*c-Jun* R	CGCAACCAGTCAAGTTCTCA		
*Egr4* F	TCTCTCCAAGCCCACCGAAG	NM_020596.3	[20,23]
*Egr4* R	AACCGCCTGGATGAAGAAGC		
*Gapdh* F	TGCACCACCAACTGCTTAGC	NM_001289726.1	[23]
*Gapdh* R	TGGCATGGACTGTGGTCATG		
*Kcc2a* F	GCCGGCTCCCGAGGGAAG	NM_001355480.1/NM_001355481.1	[38]
*Kcc2a* R	CTTCTCCGTGTCCGTGCTGTTGATG		
*Kcc2b* F	GCCACCATGCTCAACAACCT	NM_020333.2	
*Kcc2b* R	ACTGAGCAGGGAGGATACCA		
*Nrsf* F	TGAGAAACCATTTCCCCAGG	NM_011263.2	[39]
*Nrsf* R	TGAGAGCTTGAGTAAGGACAAAG		
*Sp1* F	TCTCAGGCAGGCACTATCAGCA	NM_013672.2	[40]
*Sp1* R	TTGTGATGACACCAAGCTGGC		
**Primers Used for ChIP-QPCR**			
**Primer Name**	**Sequence**	**GenBank** **Accession No.**	**Source**
AP2 sense F	CACATCTTTCTCTCAGGGGTCA	NC_000068.8	
AP2 sense R	CTAAAAATAACGGCAGGGAACTAGG		
AP2 antisense F	CTCACGCCTCCTGCATACG		
AP2 antisense R	GCTCTCTCGCTTGCTCTCC		

## Data Availability

All data are contained within the manuscript.

## Supplementary Materials

The following supporting information can be downloaded at: https://www.mdpi.com/article/10.3390/cells11233861/s1, Figure S1: Molecular markers for pre-Bötzinger complex (preBötC) neurons.

## References

[1] Rivera C., Voipio J., Payne J., Ruusuvuori E., Lahtinen H., Lamsa K., Pirvola U., Saarma M., Kaila K. (1999). The K^+^/Cl^−^ co-transporter KCC2 renders GABA hyperpolarizing during neuronal maturation. Nature.

[2] Li H., Khirug S., Cai C., Ludwig A., Blaesse P., Kolikova J., Afzalov R., Coleman S.K., Lauri S., Airaksinen M.S. (2007). KCC2 interacts with the dendritic cytoskeleton to promore spine development. Neuron.

[3] Fiumelli H., Briner A., Puskarjov M., Blaesse P., Belem B.J., Dayer A.G., Kaila K., Martin J.L., Vutskits L. (2013). An ion transport-independent role for the cation-chloride cotransporter KCC2 in dendritic spinogenesis in vivo. Cereb. Cortex.

[4] Llano O., Smirnov S., Soni S., Golubtsov A., Guillemin I., Hotulainen P., Medina I., Nothwang H.G., Rivera C., Ludwig A. (2015). KCC2 regulates actin dynamics in dendritic spines via interaction with β-PIX. J. Cell Biol..

[5] Mavrovic M., Uvarov P., Delpire E., Vutskits L., Kaila K., Puskarjov M. (2020). Loss of non-canonical KCC2 functions promotes developmental apoptosis of cortical projection neurons. EMBO Rep..

[6] Kaila K., Price T.J., Payne J.A., Puskarjov M., Voipio J. (2014). Cation-chloride transporters in neuronal development, plasticity and disease. Nat. Rev. Neurosci..

[7] Moore Y.E., Kelley M.R., Brandon N.J., Deeb T.Z., Moss S.J. (2017). Seizing control of KCC2: A new therapeutic target for epilepsy. Trends Neurosci..

[8] Rinehart J., Maksimova Y.D., Tanis J.E., Stone K.L., Hodson C.A., Zhang J., Risinger M., Pan W., Wu D., Colangela C.M. (2009). Sites of regulated phosphorylation that control K-Cl cotransporter activity. Cell.

[9] Kahle K.T., Deeb T.Z., Puskarjov M., Silayeva L., Liang B., Kaila K., Moss S.J. (2013). Modulation of neuronal activity by phosphorylation of the K-Cl cotransporter KCC2. Trends Neurosci..

[10] Lee H.H., Walker J.A., Williams J.R., Goodier R.J., Payne J.A., Moss S.J. (2007). Direct protein kinase C-dependent phosphorylation regulates the cell surface stability and activity of the potassium chloride cotransporter KCC2. J. Biol. Chem..

[11] Friedel P., Kahle K.T., Zhang J., Hertz N., Pisella L.I., Buhler E., Schaller F., Duan J., Khanna A.R., Bishop P.N. (2015). WNK1-regulated inhibitory phosphorylation of the KCC2 cotransporter maintains the depolarizing action of GABA in immature neurons. Sci. Signal..

[12] Cordshagen A., Busch W., Winklhofer M., Nothwang H.G., Hartmann A.M. (2018). Phosphoregulation of the intracellular termini of K^+^-Cl^−^ cotransporter 2 (KCC2) enables flexible control of its activity. J. Biol. Chem..

[13] Zhang J., Cordshagen A., Medina I., Nothwang H.G., Wisniewski J.R., Winklhofer M., Hartmann A.M. (2020). Staurosporine and NEM mainly impair WNK-SPAK/OSR1 mediated phosphorylation of KCC2 and NKCC1. PLoS ONE.

[14] Watanabe M., Zhang J., Mansuri M.S., Duan J., Karimy J.K., Delpire E., Alper S.L., Lifton R.P., Fukuda A., Kahle K.T. (2019). Developmentally regulated KCC2 phosphorylation is essential for dynamic GABA-mediated inhibition and survival. Sci. Signal..

[15] Pisella L.I., Gaiarsa J.L., Diabira D., Zhang J., Khalilov I., Duan J., Kahle K.T., Medina I. (2019). Impaired regulation of KCC2 phosphorylation leads to neuronal network dysfunction and neurodevelopmental pathology. Sci. Signal..

[16] Moore Y.E., Deeb T.Z., Chadchankar H., Brandon N.J., Moss S.J. (2018). Potentiating KCC2 activity is sufficient to limit the onset and severity of seizures. Proc. Natl. Acad. Sci. USA.

[17] Silayeva L., Deeb T.Z., Hines R.M., Kelley M.R., Munoz M.B., Lee H.H.C., Brandon N.J., Dunlop J., Maguire J., Davies P.A. (2015). KCC2 activity is critical in limiting the onset and severity of status epilepticus. Proc. Natl. Acad. Sci. USA.

[18] Uvarov P., Ludwig A., Markkanen M., Pruunsild P., Kaila K., Delpire E., Timmusk T., Rivery C., Airaksinen M.S. (2007). A novel N-terminal isoform of the neuron-specific K-Cl cotransporter KCC2. J. Biol. Chem..

[19] Markkanen M., Uvarov P., Airaksinen M.S. (2008). Role of upstream stimulating factors in the transcriptional regulation of the neuron-specific K-Cl cotransporter KCC2. Brain Res..

[20] Uvarov P., Ludwig A., Markkanen M., Rivera C., Airaksinen M.S. (2006). Upregulation of the neuron-specific K^+^/Cl^−^ contransporter expression by transcription factor early growth response 4. J. Neurosci..

[21] Uvarov P., Pruunsild P., Timmusk T., Airaksinen M.S. (2005). Neuronal K^+^/Cl^−^ co-transporter (KCC2) transgenes lacking neurone restrictive silencer element recapitulate CNS neurone-specific expression and developmental up-regulation of endogenous KCC2 gene. J. Neurochem..

[22] Yeo M., Berglund K.B., Augustine G., Liedtke W. (2009). Novel repression of Kcc2 transcription by REST-RE-1 controls developmental switch in neuronal chloride. J. Neurosci..

[23] Ludwig A., Uvarov P., Soni S., Thomas-Crusells J., Airaksinen M.S., Rivera C. (2011). Early growth response 4 mediates BDNF induction of potassium chloride cotransporter 2 transcription. J. Neurosci..

[24] Ludwig A., Uvarov P., Pellegrino C., Thomas-Crisells J., Schuchmann S., Saarma M., Airaksinen M.S., Rivera C. (2011). Neurtrurin evokes MAPK-dependent upregulation of Egr4 and KCC2 in developing neurons. Neural Plast..

[25] Rivera C., Li H., Thomas-Crusells J., Lahtinen H., Viitanen T., Nanobashvili A., Kokaia Z., Airaksinen M.S., Voipio J., Kaila K. (2002). BDNF-induced TrkB activation down-regulates the K^+^-Cl^−^ cotransporter KCC2 and impairs neuronal Cl^−^ extrusion. J. Cell Biol..

[26] Rivera C., Voipio J., Thomas-Crusells J., Li H., Emri Z., Sipilä S., Payne J.A., Minichiello L., Saarma M., Kaila K. (2004). Mechanism of activity-dependent downregulation of the neuron-specific K-Cl cotransporter KCC2. J. Neurosci..

[27] Krieglstein K., Zheng F., Unsicker K., Alzheimer C. (2011). More than being protective: Functional roles for TGF-β/activin signaling pathways at central synapses. Trends Neurosci..

[28] Roussa E., Wiehle M., Dünker N., Becker-Katins S., Oehlke O., Krieglstein K. (2006). Transforming growth factor beta is required for differentiation of mouse mesencephalic progenitors into dopaminergic neurons in vitro and in vivo: Ectopic induction in dorsal mesencephalon. Stem Cells.

[29] Roux J., Carles M., Koh H., Goolaerts A., Ganter M.T., Chesebro B.B., Howard M., Houseman B.T., Finkbeiner W., Shokat K.M. (2010). Transforming growth factor beta1 inhibits cystic fibrosis transmembrane conductance regulator-dependent cAMP-stimulated alveolar epithelial fluid transport via a phosphatidylinositol 3-kinase-dependent mechanism. J. Biol. Chem..

[30] Yi S., Pierucci-Alves F., Schultz B.D. (2013). Transforming growth factor-b1 impairs CFTR-mediated anion secretion across cultures porcine vas deferens epithelial monolayer via the p38 MAPK pathway. Am. J. Physiol. Cell Physiol..

[31] Kluge M., Namkoong E., Khakipoor S., Park K., Roussa E. (2019). Differential regulation of vacuolar H^+^-ATPase subunits by transforming growth factor-β1 in salivary ducts. J. Cell. Physiol..

[32] Khakipoor S., Ophoven C., Schrödl-Häußel M., Feuerstein M., Heimrich B., Deitmer J.W., Roussa E. (2017). TGF-β signaling directly regulates transcription and functional expression of the electrogenic sodium bicarbonate cotransporter 1, NBCe1 (SLC4A4), via Smad4 in mouse astrocytes. Glia.

[33] Roussa E., Speer J.M., Chudotvorova I., Khakipoor S., Smirnov S., Rivera C., Krieglstein K. (2016). The membrane trafficking and functionality of the K^+^-Cl^−^ co-transporter KCC2 is regulated by TGF-β2. J. Cell Sci..

[34] Sanford L.P., Ormsby I., Gittenberger-de Groot A.C., Sariola H., Friedman R., Boivin G.P., Cardell E.L., Doetschman T. (1997). TGFbeta2 knockout mice have multiple developmental defects that are non-overlapping with other TGFbeta knockout phenotypes. Development.

[35] Inman G.J., Nicolás F.J., Callahan J.F., Harling J.D., Gaster L.M., Reith A.D., Laping N.J., Hill C.S. (2002). SB-431542 is a potent and specific inhibitor of transforming growth factor-beta superfamily type I activin receptor-like kinase (ALK) receptors ALK4, ALK5, and ALK7. Mol. Pharmacol..

[36] Huang Z., Xu H., Sandell L. (2004). Negative Regulation of Chondrocyte Differentiation by Transcription Factor *AP-2α*. J. Bone Miner. Res..

[37] Schulien I., Hockenjos B., Schmitt-Graeff A., Perdekamp M.G., Follo M., Thimme R., Hasselblatt P. (2019). The transcription factor c-Jun/AP-1 promotes liver fibrosis during non-alcoholic steatohepatitis by regulating Osteopontin expression. Cell Death Differ..

[38] Markkanen M., Ludwig A., Khirug S., Pryazhnikov E., Soni S., Khiroug L., Delpire E., Rivera C., Airaksinen M.S., Uvarov P. (2017). Implications of the N-terminal heterogeneity for the neuronal K-Cl cotransporter KCC2 function. Brain Res..

[39] Li H., Liu Z., Wu Y., Chen Y., Wang J., Wang Z., Huang D., Wang M., Yu M., Fei J. (2020). The deficiency of NRSF/REST enhances the pro-inflammatory function of astrocytes in a model of Parkinson’s disease. Biochim. Biophys. Acta (BBA)-Mol. Basis Dis..

[40] García-Morales V., Rodríguez-Bey G., Gómez-Pérez L., Domínguez-Vías G., González-Forero D., Portillo F., Campos-Caro A., Gento-Caro Á., Issaoui N., Soler R.M. (2019). Sp1-regulated expression of p11 contributes to motor neuron degeneration by membrane insertion of TASK1. Nat. Commun..

[41] Chleilat E., Pethe A., Pfeifer D., Krieglstein K., Roussa E. (2020). TGF-β signaling regulates SLC8A3 expression and prevents oxidative stress in developing midbrain dopaminergic and dorsal raphe serotonergic neurons. Int. J. Mol. Sci..

[42] Ludwig A., Li H., Saarma M., Kaila K., Rivera C. (2003). Developmental up-regulation of KCC2 in the absence of GABAergic and glutamatergic transmission. Eur. J. Neurosci..

[43] Feng X.H., Lin X., Derynck R. (2000). Smad2, Smad3 and Smad4 cooparate with Sp1 to induce p15(Ink4B) transcription in response to TGF-beta. EMBO J..

[44] Koinuma D., Tsutsumi S., Kamimura N., Taniguchi H., Miyazawa K., Sunamura M., Imamura T., Miyazono K., Aburatani H. (2009). Chromatin immunoprecipitation on Microarray analysis of Smad2/3 binding sites reveals roles of ETS1 and TFAP2A in transforming growth factor b signaling. Mol. Cell Biol..

[45] Yao C.D., Haensel D., Gaddam S., Patel T., Atwood S.X., Sarin K.Y., Whitson R.J., McKellar S., Shankar G., Aasi S. (2020). AP-1 and TGF-b cooperativity drives non-canonical Hedgehog signaling in resistant basal cell carcinoma. Nat. Commun..

[46] Smith J.C., Ellenberger H.H., Ballanyi K., Richter D.W., Feldman J.L. (1991). Pre-Bötzinger complex: A brainstem region that may generate respiratory rhythm in mammals. Science.

[47] Hübner C.A., Stein V., Hermans-Borgmeyer I., Meyer T., Ballanyi K., Jentsch T.J. (2001). Disruption of KCC2 reveals an essential role of K-Cl cotransport already in early synaptic inhibition. Neuron.

[48] Koizumi H., Koshiya N., Chia X.J., Cao F., Nugent J., Zhang R., Smith J.C. (2013). Structural-functional properties of identified excitatory and inhibitory interneurons within Pre-Bötzinger complex respiratory microcircuits. J. Neurosci..

[49] Heupel K., Sargsyan V., Plomp J.J., Rickmann M., Varoqueaux F., Zhang W., Krieglstein K. (2008). Loss of transforming growth factor-beta 2 leads to impairment of central synapse function. Neural Dev..

[50] Stornetta R.L., Rosin D.L., Wang H., Sevigny C.P., Weston M.C., Guyenet P.G. (2003). A group of glutamatergic interneurons expressing high levels of both neurokinin-1 receptors and somatostatin identifies the region of the pre-Bötzinger complex. J. Comp. Neurol..

[51] Thoby-Brisson M., Karlen M., Wu N., Charnay P., Champagnat J., Fortin G. (2009). Genetic identification of an embryonic parafacial oscillator coupling to the preBotzinger complex. Nat. Neurosci..

[52] Balakrishnan V., Becker M., Löhrke S., Nothwang H.G., Güresir E., Friauf E. (2003). Expression and function of chloride transporters during development of inhibitory neurotransmission in the auditory brainstem. J. Neurosci..

[53] Khiroug L., Huttu K., Ludwig A., Smirnov S., Voipio J., Rivera C., Kaila K., Khiroug L. (2005). Dinstinct properties of functional KCC2 expression in immature mouse hippocampal neurons in culture and in acute slices. Eur. J. Neurosci..

[54] Aguado F., Carmona M.A., Pozas E., Aguilo A., Martinez-Guijarro F.J., Alcantara S., Borrell V., Yuste R., Ibañez C.F., Soriano E. (2003). BDNF regulates spontaneous correlated activity at early developmental stages by increasing synaprogenesis and expression of the K^+^/Cl^−^ co-transporter KCC2. Development.

[55] Carmona M.A., Pozas E., Martinaz A., Espinoza-Parrilla J.F., Soriano E., Aguado F. (2006). Age-dependent spontaneous hyperexcitability and impairment of GABAergic function in the hippocampus of mice lacking trkB. Cereb. Cortex.

[56] Puskarjov M., Ahmad F., Khirug S., Sivakumaran S., Kaila K., Blaesse P. (2015). BDNF is required for seizure-induced but not developmental up-regulation of KCC2 in the neonatal hippocampus. Neuropharmacology.

