# Supplementary material for: TGF-β2 Regulates Transcription of the K+/Cl− Cotransporter 2 (KCC2) in Immature Neurons and Its Phosphorylation at T1007 in Differentiated Neurons
Source: Cells. 2022 Nov 30;11(23):3861. doi: 10.3390/cells11233861 (PMC9739967; doi:10.3390/cells11233861)
Supplement: Supplementary file 1 [file cells-11-03861-s001.zip › Caption Figure S1.pdf]

Figure S1: Molecular markers for pre-Bötzinger complex (preBötC) neurons. (A) Visualization of preBötC by double immunofluorescence for somatostatin (SST; red) and the neurokinin 1 receptor (NK1R; green) on fixed mouse brain cryosections. The preBötC with its NKR1+/SST+/Phox2b- "core" neurons is bordered by the red dotted circle, yellow dotted circle includes neurons of the nucleus ambiguus (NK1R+/SST-/Phox2b+), and cells of the facial nerve (N7, nucleus of the facial nerve) are bordered by the blue dotted circle (NK1R-/SST-/Phox2b+). **(**B) Immunolabeling for Phox2b applied to distinguish preBötC from neighbouring nuclei. Scale bar: 200μm
