# Supplementary figures and images for: TGF-β2 Regulates Transcription of the K+/Cl− Cotransporter 2 (KCC2) in Immature Neurons and Its Phosphorylation at T1007 in Differentiated Neurons
Source: Cells. 2022 Nov 30;11(23):3861. doi: 10.3390/cells11233861 (PMC9739967; doi:10.3390/cells11233861)

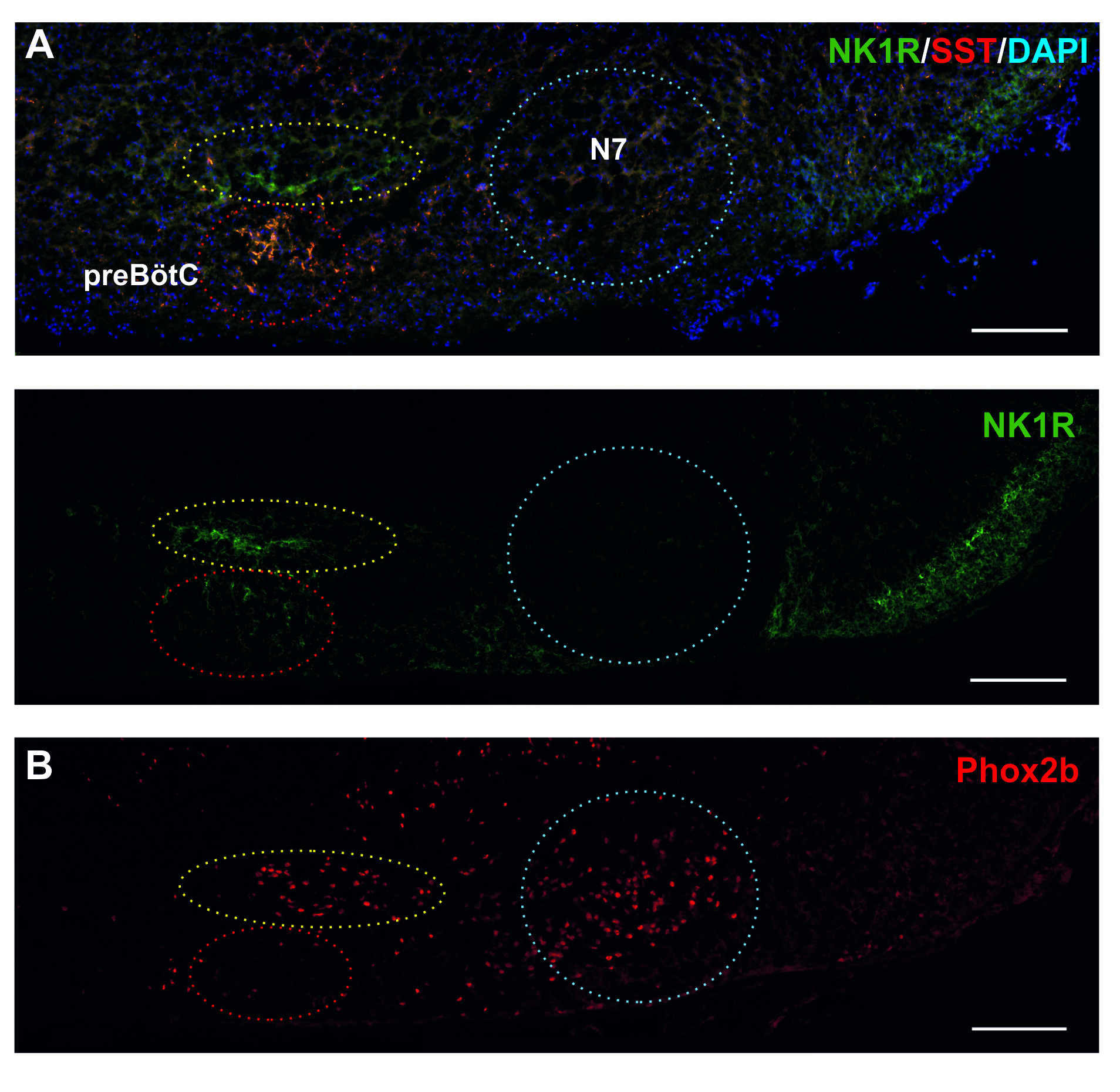

Supplement: Supplementary file 1 [file cells-11-03861-s001.zip › Supplementary figure S1.jpg]
